# A Particle-Based Cohesive Crack Model for Brittle Fracture Problems

**DOI:** 10.3390/ma13163573

**Published:** 2020-08-13

**Authors:** Hu Chen, Y. X. Zhang, Linpei Zhu, Fei Xiong, Jing Liu, Wei Gao

**Affiliations:** 1 GAC R&D Center, Guangzhou Automobile Group CO., LTD, Guangzhou 511434, China; huchen.unsw@outlook.com (H.C.); zhulinpei@gacrnd.com (L.Z.); xiongfei@gacrnd.com (F.X.); liujing@gacrnd.com (J.L.); 2 School of Engineering, Western Sydney University, Sydney, NSW 2751, Australia; 3 School of Electromechanical Engineering, Guangdong University of Technology, Guangzhou 510006, China

**Keywords:** discrete element, cohesive crack model, brittle fracture, mixed-mode fracture

## Abstract

Numerical simulations of the fracture process are challenging, and the discrete element (DE) method is an effective means to model fracture problems. The DE model comprises the DE connective model and DE contact model, where the former is used for the representation of isotropic solids before cracks initiate, while the latter is employed to represent particulate materials after cracks propagate. In this paper, a DE particle-based cohesive crack model is developed to model the mixed-mode fracture process of brittle materials, aiming to simulate the material transition from a solid phase to a particulate phase. Because of the particle characteristics of the DE connective model, the cohesive crack model is constructed at inter-particle bonds in the connective stage of the model at a microscale. A potential formulation is adopted by the cohesive zone method, and a linear softening relation is employed by the traction–separation law upon fracture initiation. This particle-based cohesive crack model bridges the microscopic gap between the connective model and the contact model and, thus, is suitable to describe the material separation process from solids to particulates. The proposed model is validated by a number of standard fracture tests, and numerical results are found to be in good agreement with the analytical solutions. A notched concrete beam subjected to an impact loading is modeled, and the impact force obtained from the numerical modeling agrees better with the experimental result than that obtained from the finite element method.

## 1. Introduction

Fracture is a common failure mode for engineering structures and structural components. When structures are subjected to severe loading or large deformation, new fracture surfaces are created and cracks occur. Effective numerical modeling of the fracture process is important for assessing the safety, reliability, and structural performance of engineering structures. For modeling the fracture process, a number of numerical methods are often employed, such as the finite element method (FEM) [1], the extended finite element method (XFEM) [2], meshless methods [3], the particle finite element method (PFEM) [4], molecular dynamics [5], the particle method [6], and atomistic methods [7,8]. Among them, the atomistic method is able to provide great insight into the nanoscopic mechanism of fracture initiation and propagation since fracture problems essentially take place at the atomic level of materials by means of the breakage of bonds [9,10]. Modeling at the atomistic scale, however, is computationally intensive because extremely small length and time scales have to be adopted [11]. Therefore, atomistic modeling is generally not applicable to practical engineering problems.

The discrete element method (DEM) [12] describes particle characteristics, and it is widely applied to model the fracture behavior of brittle and quasi-brittle materials, such as glass [13,14], ceramics [15,16], and concrete [17,18]. The conventional DE model is based on contact interaction. It should be noted that DE particles can also be connected by springs and assembled in a lattice structure [19,20,21]. The DE lattice model is able to represent continua in its connective form (i.e., connective model), and it can describe discontinua by means of contact (i.e., contact model). The representation of continua by the connective model is based on the energy equivalence between the strain energy stored in solids and that stored in bonding springs. Using this connective model, the macro-structural material response can be determined by the micromechanical interaction between particles at microscale, which is essentially different from the phenomenological material constitutive law often adopted by the FEM. Moreover, this connective model is able to deal with fracture by means of the breakage of bonding springs. Compared with the atomistic methods, the DE connective model describes the same features of particle characteristics and the breakage of bonds. Importantly, the DEM has much larger length and time scales allowing the computation to be much more efficient.

When fracture occurs in engineering materials, a small process zone exists ahead of the crack tip, where micro-cracking, void nucleation, growth, and coalescence might take place [22]. The most common tool to formulate such a fracture process zone is the cohesive zone method (CZM), which can be traced back to the work by Dugdale [23] and Barenblatt [24], and it was applied for modeling both brittle materials [25,26] and ductile materials [27]. CZMs describe the cohesive relation between the traction and the separation of newly created fracture surfaces in the process zone. When used along with the FEM, the cohesive elements are embedded into FE boundaries, and fracture initiation and propagation are described by the evolution of a cohesive zone. In general, CZMs are categorized into intrinsic ones and extrinsic ones. In the intrinsic CZM [1,14,28,29,30], the interfacial cohesive elements need to be pre-defined in the possible fracture paths. It should be noted that the cohesive law in intrinsic CZMs consists of an initial loading stage and a softening stage, where the former stage is unphysical and introduces artificial compliance into the model. The determination of the initial loading stiffness is troublesome. If the stiffness is too small, it may lead to inaccurate fracture behavior due to the introduced artificial compliance; however, if it is too large, it may reduce the size of the time step and cause computational instability. By contrast, the extrinsic CZM [31,32,33] does not need pre-defined cohesive elements and, thus, the original model remains intact until cracks occur at the onset of fracture. Furthermore, the extrinsic CZM avoids the problem of seeking an appropriate initial loading stiffness, but a complicated data structure is needed to organize the newly created cohesive surfaces. The CZM associated with the DE connective model is able to get rid of the drawbacks mentioned above. This is due to the DE connective model, which is initially bonded by springs, while the spring connection and stiffness can be directly used in the initial loading stage. Moreover, cracks occur by means of the breakage of bonding springs rather than the split of particles and, thus, the complicated data structure is not needed. 

In this paper, a particle-based cohesive crack model is proposed for modeling the mixed-mode fracture process of brittle and quasi-brittle materials. The DE connective model is used to represent homogeneous and isotropic solids at microscale, and the DE contact model is used to describe the particulate materials of discontinua. DE particles change the model from a connective one to a contact one due to the breakage of bonding springs. Accordingly, material transition takes place from a solid phase to a particulate phase. Since such a perfectly brittle material does not exist, there might be an intermediate phase between the solid phase and the particulate phase where little deformation occurs. A CZM is then incorporated into the springs of the DE connective model, and the CZM is used to describe the cohesive transition phase so as to model the fracture process. Each particle pair in the DE connective model constitutes a cohesive element and, thus, the CZM is formulated at the same scale of the connective model. A potential formulation is adopted for the CZM, and a linear softening relation is employed for the traction–separation law upon fracture initiation. The criteria of both the fracture initiation and the fracture propagation are constructed in the displacement space. A damage parameter is used to record the damage state and, thus, the fracture process is irreversible. Note that some CZMs are also used in conjunction with the DEM for fracture modeling in homogeneous materials [18,34,35,36] and laminated materials [30,37,38], but the cohesive formulation differs.

The remainder of the paper is organized as follows: the DE connective model is firstly outlined to describe the intact stage of elastic solids in Section 2. Then, a particle-based cohesive crack model is proposed in Section 3 to detail the fracture process of materials, where mixed-mode fracture initiation and propagation criteria are derived. Once the decohesion is completed, the conventional DE contact model is applied to describe the interaction of DE particles in Section 4. The model transition process and the implementation issue of the model are discussed in Section 5 and Section 6, respectively. To validate the proposed model, a number of numerical examples are presented in Section 7. Lastly, some conclusions are drawn.

## 2. Connective Model: Representation of Isotropic Elastic Solid

The DE connective method is favorable for modeling elastic–brittle materials, such as glass [13], because this model can be conveniently switched to the contact model. As the DE connective model is capable of modeling the response of materials which are initially continuous but eventually cracked [19], this model is employed herein to represent the elastic continuum used in the domain of particular interest. It is worth noting that, in most DE connective models, all DE particles have the same geometrical shape and size for easy characterization of material properties.

**Figure 1:**
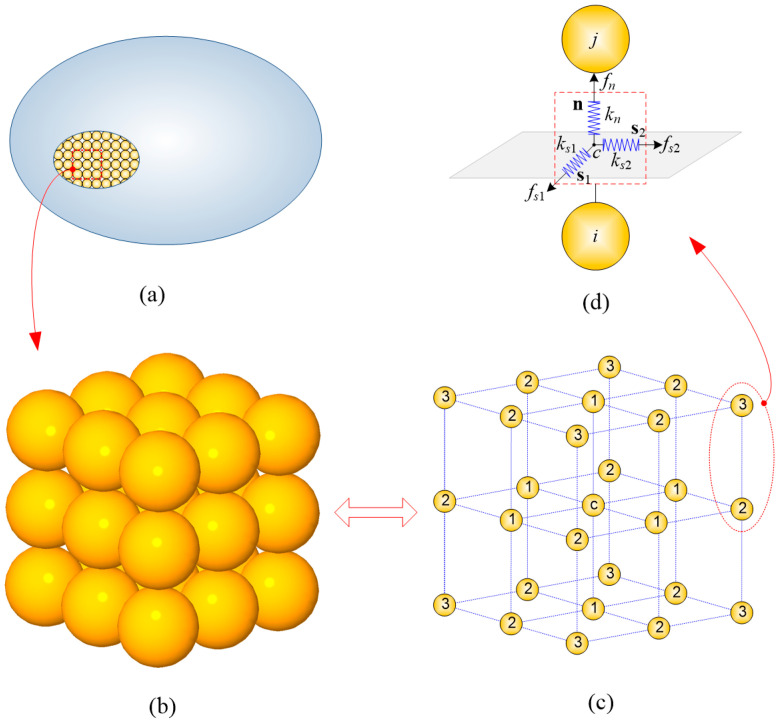
Discrete representation of an elastic isotropic solid: (**a**) solids represented by the discrete element model; (**b**) the connective model of discrete elements and the unit cell of 27 spherical particles located in a cubic structure; (**c**) three types of linking relation between particles; (**d**) virtual springs connecting adjacent particles.

In the DE connective model shown in Figure 1, spherical particles and virtual springs are used at microscale to formulate homogeneous and isotropic solids, generally described by the FEM using a phenomenological material constitutive law at macroscale. The formulation is based on the energy equivalence, i.e., the energy stored in springs is equal to that stored in elastic solids. The spring stiffness is derived based on the Cauchy–Born rule [39], and the details of this derivation are given in Reference [40].

A number of DE connective models were reported, for which the unit cell is either in a hexagonal structure [18,19,41] or in a cubic structure [20,41,42,43]. In a hexagonal structure, the DE particles are stacked in a denser manner; however, the boundary of the model is not flat and, thus, not favorable for the coupling approach with the FEM. The cubic model proposed by Yu [20] was employed because of its computational accuracy and unique structure. This particular structure can result in flat boundaries with the DE model, which is practically very desirable for domain decomposition of coupling models because the burden of pre-processing can be effectively eased. 

The unit cell structure of this model is shown in Figure 1b. It is apparent that this model is composed of 27 spherical particles of the same size which are located in a cubic structure. The central particle of this model is connected to 26 neighboring particles, which can be categorized into three groups according to their distance to the central one, as depicted by different numbers in Figure 1c. The interaction force ${\hat{f}}^{\mathrm{int}}$ between any two adjacent particles in the local coordinate system is calculated based on their relative displacement δ and spring stiffness K (see Figure 1d) as follows:

(1) $${\hat{f}}^{\mathrm{int}}=K\delta ,$$

where force ${\hat{f}}^{\mathrm{int}}={\left[{\hat{f}}_{n}\;{\hat{f}}_{s1}\;{\hat{f}}_{s2}\right]}^{T}$ and $\delta ={\left[\delta_{n},{\;\delta }_{s1},{\;\delta }_{s2}\right]}^{T}$, while K is given as follows:

(2) $$K=\left[\begin{array}{ccc} k_{n} & 0 & 0\\ 0 & k_{s1} & 0\\ 0 & 0 & k_{s2} \end{array}\right]$$

Inside each pair, as shown in Figure 1d, there are one orthogonal ($k_{n}$) and two tangential ($k_{s1}$ and $k_{s2}$) linear springs invisibly connecting them. Note that subscripts n, s1, and s2 denote the unit directions of the local coordinate, as shown in Figure 1d. Their stiffness is determined based on the energy equivalence between that stored in the springs and that stored in solid elasticity, as given by Equation (3) [20].

(3) $$\{\begin{array}{c} k_{n}^{1}=k_{n}^{2}=\frac{2Er}{5\left(1-2\nu \right)}\\ k_{s1}^{1}=k_{s2}^{1}=k_{s1}^{2}=k_{s2}^{2}=\frac{2Er\left(1-4\nu \right)}{5\left(1-2\nu \right)\left(1+\nu \right)}\\ k_{n}^{3}=k_{s1}^{3}=k_{s2}^{3}=0 \end{array},$$

where E, ν, and r are the Young’s modulus, Poisson’s ratio, and the radius of DE particles, respectively. Note that superscripts in Equation (3) denote the type of connecting springs between each particle pair, as shown in Figure 1c.

Let n, $s_{1}$, and $s_{2}$ be the unit base vectors of the local coordinate system to be expressed in the global coordinate system, which is shown in Figure 1d. Then, the transformation matrix ϕ from the global frame to the local frame can be expressed by

(4) $$\phi =\left[\begin{array}{ccc} n_{1} & n_{2} & n_{3}\\ s_{1}^{1} & s_{2}^{1} & s_{3}^{1}\\ s_{1}^{2} & s_{2}^{2} & s_{3}^{2} \end{array}\right],$$

where $n_{1}$, $n_{2}$, and $n_{3}$ are the components of n, while $s_{1}^{1}$, $s_{2}^{1}$, and $s_{3}^{1}$ are the components of $s_{1}$; note that, here, the subscript replaced by superscript for clarity. The same interpretation and treatment of $s_{1}$ apply to $s_{2}$.

Therefore, the internal force $f^{\mathrm{int}}$ in the global coordinate system can be obtained by

(5) $$f^{\mathrm{int}}=\phi^{T}{\hat{f}}^{\mathrm{int}},$$

and the moment $m^{\mathrm{int}}$ is calculated as

(6) $$m^{\mathrm{int}}=\bar{r}\times f^{\mathrm{int}},$$

where $\bar{r}$ denotes the effective radius vector, originating from the particle’s center to the middle point between the two particles of a pair. Taking Figure 1d as an example, the effective radius vector for the particle i is ${\bar{r}}_{i}=x_{c}-x_{i}$, where $x_{c}$ and $x_{i}$ denote position vectors of the particle i and middle point c, respectively.

Based on Equation (1), the relation between the traction $T={\left[T_{n},{\;T}_{s1},{\;T}_{s2}\right]}^{T}$ and the relative displacement δ can be written as

(7) $$T_{n}=k_{n}\delta_{n}/A,$$

(8) $$T_{s1}=k_{s1}\delta_{s1}/A,$$

(9) $$T_{s2}=k_{s2}\delta_{s2}/A,$$

where $T_{n}$, $T_{s1}$, and $T_{s2}$ are the traction components along the normal and the two shear directions in the local frame, respectively, $\delta_{n}$, $\delta_{s1}$, and $\delta_{s2}$ are the relative displacement components (or separation components) along the normal and the two shear directions, respectively, and A is an effective area between a particle pair; for the first and second nearest particle pairs, it is $\;\pi r^{2}/4$ and $2\pi r^{2}/9$, respectively [40]. Note that, as the spring stiffness of the third type of connecting particle pair is zero as shown in Equation (3), the effective area of this type is not useful and, thus, ignored.

Since spring stiffness in two shear directions are the same, as seen in Equation (3), i.e., $k_{s}=k_{s1}=k_{s2}$, the two relative displacement components $\delta_{s1}$ and $\delta_{s2}$ can be considered together as

(10) $$\delta_{s}=\sqrt{\delta_{s1}^{2}+\delta_{s2}^{2}}.$$

Then, Equations (8) and (9) can be combined as

(11) $$T_{s}=k_{s}\delta_{s}/A.$$

**Figure 2:**
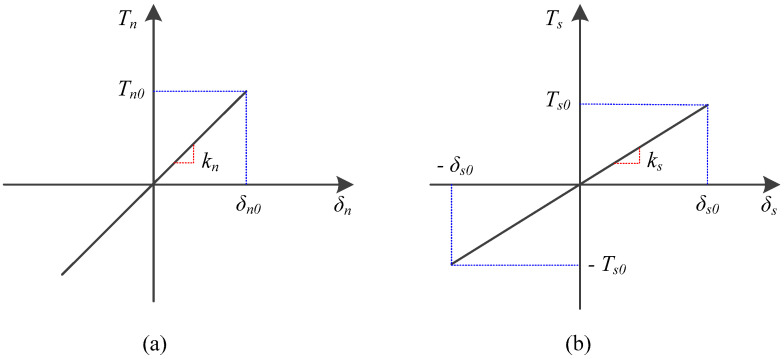
Linear relation between traction and relative displacement in (**a**) the normal direction and (**b**) the shear direction.

The linear relation between the traction and relative displacement are illustrated in Figure 2. 

The critical values of the relative displacement at the elastic limit, $\delta_{n0}$ and $\delta_{s0}$, can be calculated as

(12) $$\delta_{n0}=AT_{n0}/k_{n},$$

(13) $$\delta_{s0}=AT_{s0}/k_{s},$$

where $T_{n0}$ and $T_{s0}$ are the material strengths in the normal and shear directions, respectively. For isotropic solids, the material strengths are assumed to be the same, i.e., $T_{n0}=T_{s0}=\sigma_{c}$.

## 3. Cohesive Crack Model: Formulation of Fracture Process

### 3.1. General Description

**Figure 3:**
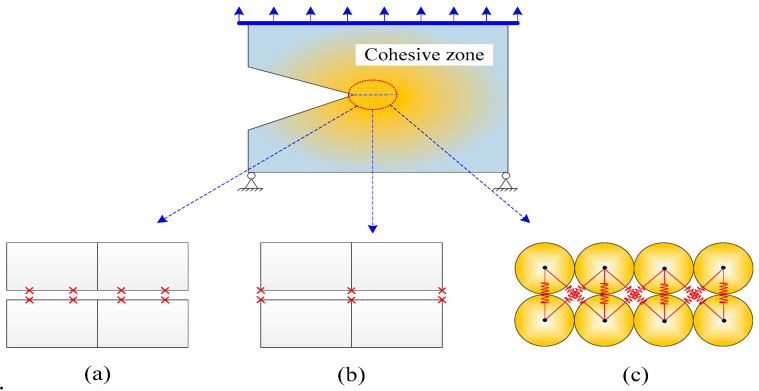
Schematic of different cohesive zone models in two dimensions: (**a**) continuum cohesive zone model with interaction through quadrature points; (**b**) discrete cohesive zone model with interaction through nodes; (**c**) particle-based cohesive zone model with interaction through DE particles.

The general CZM used in the FEM is formulated between the FE surfaces in three dimensions. The FE formulation of the cohesive zone has two different forms in general, the widely used continuum CZM [28,31,44] as shown in Figure 3a and the discrete CZM [45,46,47] as shown in Figure 3b. The continuum CZM treats the fracture process zone between any two continuous FE surfaces as a cohesive element, and the cohesive traction is related to the displacement jump between the quadrature points on the separate surfaces by means of a defined cohesive law. Therefore, this cohesive formulation is surface-wise, and the cohesive traction between each quadrature point pair is dependent on the nodal displacements of the cohesive element. This surface-wise cohesive formulation may suffer from non-convergence [48], and great care must be taken with the numerical integration scheme for the cohesive elements [49,50]. By contrast, the discrete CZM constructs cohesive elements at adjacent nodes by means of a nonlinear spring [48]. The cohesive traction of this discrete spring-type formulation depends only on the displacement jump between the node pair and, thus, this cohesive formulation is point-wise. In contrast to the surface-wise cohesive elements, the point-wise cohesive elements have better convergence and are insensitive to mesh size and loading rate.

In line with the point-wise cohesive formulation, the cohesive zone associated with the lattice DE model is inserted into each adjacent particle pair, shown in Figure 3c as a bridging zone between the DE connective model and the DE contact model. Each particle pair of the connective model constitutes a cohesive element and, thus, the CZM is formulated at microscale. It is evident from Figure 3c that the cohesive traction is only related to the displacement jump of the particle pair. Note that Gao and Klein [51] also used a cohesive formulation for material particles at microscale, but their formulation relied on a virtual internal bond model.

As shown in Figure 3c, the connection orientations of the particle-based CZM vary from pair to pair and, thus, the opening directions of crack fronts can never be uniform at microscale, which is different from the case of the continuum CZM (Figure 3a) and the discrete CZM (Figure 3b).

A mixed-mode propagation criterion for the particle-based CZM is firstly proposed to describe the model transition from a cohesive model to a contact model, and then a mixed-mode initiation criterion is proposed for the model transition from a connective model to a cohesive model.

### 3.2. Mixed-Mode Fracture Propagation Criterion

Based on the fracture model originally developed by Tvergaard and Hutchinson [52] and modified by Espinosa and Zavattieri [53] and Song et al. [54], a potential-based mixed-mode formulation is proposed for the particle-based CZM at microscale. To formulate this mixed-mode cohesive relation, a non-dimensional scalar λ of the effective displacement jump is defined as

(14) $$\lambda =\sqrt{{\left(\frac{\delta_{n}}{\delta_{nc}}\right)}^{2}+{\left(\frac{\delta_{s}}{\delta_{sc}}\right)}^{2}},$$

where <■> is the Macaulay bracket defined by <■>=((■)+|■|)/2, $\delta_{n}$ and $\delta_{s}$ are current opening and shear separation components, and $\delta_{\mathrm{nc}}$ and $\delta_{\mathrm{sc}}$ are critical values for opening mode and shear mode at complete cracking points, respectively. Equation (14) indicates that both opening displacement and shear separation displacement are taken into account for the evaluation of fracture propagation initiation. Note that the use of the Macaulay bracket on the opening crack indicates that only the tensional fracture is accounted for, while the fracture due to compression is not considered in the present cohesive law. The resistance to compression is formulated as a penalty-like force with $k_{n}$ as the penalty stiffness.

**Figure 4:**
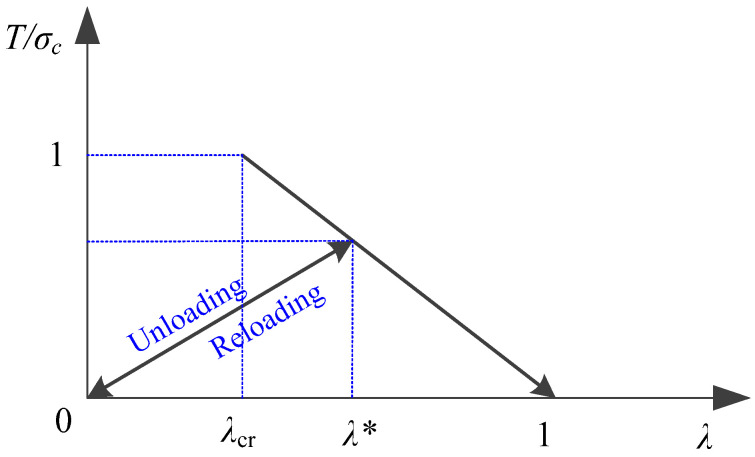
Linear traction–separation law.

An effective traction T(λ) is constructed in the form of linear softening as follows:

(15) $$T\left(\lambda \right)=\sigma_{c}\frac{\left(1-\lambda \right)}{\left(1-\lambda_{cr}\right)},$$

where the cohesive strength $\sigma_{c}$ is the material tensile strength. As illustrated in Figure 4, this traction reduces linearly from the material cohesive strength at the fracture initiation point $\lambda_{\mathrm{cr}}$ to zero at the decohesion point when Equation (16) is satisfied.

(16) $$\sqrt{{\left(\frac{\delta_{n}}{\delta_{nc}}\right)}^{2}+{\left(\frac{\delta_{s}}{\delta_{sc}}\right)}^{2}}=1.$$

The fracture initiation point $\lambda_{\mathrm{cr}}$ denotes a critical value of the effective displacement jump at which the effective traction  T(λ) reaches the maximum. $\lambda_{\mathrm{cr}}$ is determined by the initiation criterion introduced in next section. Note that the critical value $\lambda_{\mathrm{cr}}$ is dependent on the physical parameters of the particle-based CZM rather than a user-defined value as used to adjust initial loading stiffness [53,54].

Using the effective displacement jump and the effective traction, a potential function Φ(λ) is constructed as follows [52]:

(17) $$\Phi \left(\lambda \right)=\delta_{nc}\int_{\lambda_{cr}}^{\lambda }T\left(\lambda^{\prime }\right)d\lambda^{\prime }.$$

Furthermore, the total dissipated energy within the linear softening stage per unit area of newly created fracture surfaces is defined as the critical energy release rate $G_{c}$, which is given by

(18) $$G_{c}=\delta_{nc}\int_{\lambda_{cr}}^{1}T\left(\lambda^{\prime }\right)d\lambda^{\prime }.$$

The first derivative of the potential function yields the opening component $T_{n}$ and shear component $T_{s}$ of the cohesive traction as follows:

(19) $$T_{n}=\frac{\partial \Phi }{\partial \delta_{n}}=\frac{\partial \Phi }{\partial \lambda }\frac{\partial \lambda }{\partial \delta_{n}}=\frac{T\left(\lambda \right)\;}{\lambda }\frac{\delta_{n}}{\delta_{nc}}$$

(20) $$T_{s}=\frac{\partial \Phi }{\partial \delta_{s}}=\frac{\partial \Phi }{\partial \lambda }\frac{\partial \lambda }{\partial \delta_{s}}=\frac{T\left(\lambda \right)\;}{\lambda }\frac{\delta_{nc}}{\delta_{sc}}\frac{\delta_{s}}{\delta_{sc}}.$$

Apparently, both traction components, $T_{n}$ and $T_{s}$, are dependent on two separation components, $\delta_{n}$ and $\delta_{s}$. When λ=1, both components vanish without any extra enforcements, indicating that the decohesion at both modes is completed simultaneously. This is consistent with the physical phenomenon [55]. Note that this particular feature is sought in formulating the initiation criterion in next section. Once λ≥1, fracture propagates to the next particle pair, and the current particle pair is transitioned to be in a contact condition. The specific treatment on contact is illustrated in Section 4.

Since the fracture process is irreversible, the irreversibility of the cohesive law is realized by recording the maximum λ as

(21) $$\lambda^{*}=\underset{0<t^{\prime }<t}{\max}\left\{\lambda \left(t^{\prime }\right),\;\lambda \left(t\right)\right\}.$$

The damage can then be defined by a scalar 𝒹 as

(22) $$𝒹=\frac{\lambda^{*}-\lambda_{cr}}{1-\lambda_{cr}}.$$

As $\lambda^{*}$ is monotonically increasing, the history of damage, as shown by Equations (21) and (22), is monotonic as well, and this is consistent with the damage mechanics. This damage definition provides a good description of the damage state as the damage process varies in a linear form, starting from 0 for a state without damage to 1 for a state with full damage [56].

The unloading is assumed to be back to the origin as shown in Figure 4, and the reloading is assumed to be back to the original point as determined by $\lambda^{*}$ without any energy dissipation. During the course of unloading/reloading, the loading ratio 𝓇 is defined as

(23) $$𝓇=\frac{\lambda }{\lambda^{*}}.$$

Then, the effective traction can be generally rewritten as

(24) $$T\left(\lambda \right)=\sigma_{c}\left(1-𝒹\right)𝓇.$$

It is apparent that the effective traction relies on the damage history as defined by Equation (23). Furthermore, the traction components as shown in Equation (19) can be rewritten in general form as follows:

(25) $$T_{n}=\frac{T(\lambda )}{\lambda^{*}}\frac{\delta_{n}}{\delta_{nc}}=\sigma_{c}\left(1-𝒹\right)𝓇\frac{1}{\lambda^{*}}\frac{\delta_{n}}{\delta_{nc}},$$

(26) $$T_{s}=\frac{T(\lambda )}{\lambda^{*}}\frac{\delta_{nc}}{\delta_{sc}}\frac{\delta_{s}}{\delta_{sc}}=\sigma_{c}\left(1-𝒹\right)𝓇\frac{1}{\lambda^{*}}\frac{\delta_{nc}}{\delta_{sc}}\frac{\delta_{s}}{\delta_{sc}}.$$

Therefore, two different shear traction components can be determined by

(27) $$T_{s1}=T_{s}\frac{\delta_{s1}}{\delta_{s}}\;\mathrm{and}\;\;T_{s2}=T_{s}\frac{\delta_{s2}}{\delta_{s}}.$$

Note that these traction components are determined in the local frame.

The traction vector T is used to determine the local cohesive force ${\hat{f}}^{\mathrm{coh}}$ as

(28) $${\hat{f}}^{\mathrm{coh}}=AT.$$

The cohesive force $f^{\mathrm{coh}}$ at the global frame can then be determined using the transformation matrix ϕ Equation (4) as

(29) $$f^{\mathrm{coh}}=\Phi^{T}{\hat{f}}^{\mathrm{coh}}.$$

Moreover, the moment $m^{\mathrm{coh}}$ related to the cohesive force is obtained as

(30) $$m^{\mathrm{coh}}=\bar{r}\times f^{\mathrm{coh}},$$

where $\bar{r}$ denotes an effective radius vector.

One limitation of the cohesive law is that the critical energy release rates of the opening mode $G_{\mathrm{Ic}}$ and the shear mode $G_{\mathrm{IIc}}$ are required to be the same in any mixed-mode conditions [52], i.e., $G_{\mathrm{Ic}}=G_{\mathrm{IIc}}=G_{c}$. This is because a single $G_{c}$ is used to define the critical energy release rate as shown in Equation (18). Note that the current DE model is only applicable to homogeneous and isotropic elastic solids. For isotropic materials, the critical energy release rates at the two modes can be reasonably assumed to be the same [55,57,58]. For an extension to two different critical energy release rates, the reader can refer to Reference [53].

### 3.3. Mixed-Mode Fracture Initiation Criterion

Analogous to the formulation of the propagation criterion, a non-dimensional scalar ψ, which is similar to the scalar λ in Equation (14), is defined as

(31) $$\psi =\sqrt{{\left(\frac{\delta_{n}}{\delta_{n0}}\right)}^{2}+{\left(\frac{\delta_{s}}{\delta_{s0}}\right)}^{2}}.$$

**Figure 5:**
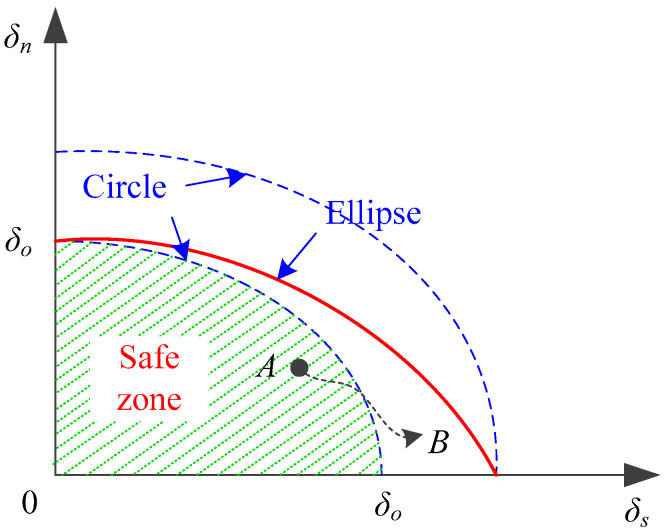
Schematic of fracture initiation criterion and the change of the mixed-mode ratio.

It is used to develop a mixed-mode initiation criterion for the onset of fracture initiation in the displacement space. Once ψ=1, fracture initiates, and Figure 5 describes this ellipse initiation criterion.

This initiation criterion was also used by Alfano and Crisfield [55], Mi et al. [57], and Qiu et al. [59] to design a mixed-mode fracture model with the intention of avoiding the pre-solution of the mixed-mode ratio $\delta_{s}∕\delta_{n}$. A pre-solution of the mode ratio or a known mode ratio is generally needed by a number of mixed-mode formulations [56,60,61,62]. These formulations are based on the energy release rate and use the power law criteria [63,64] or the B–K criterion [65] as the fracture propagation criterion. This kind of formulation also requires the mode ratio to be constant during the course of the fracture process. The mode ratio, however, changes along the fracture process zone [62] or throughout the loading [66], which might be due to the displacement jump [67] or impact events [68].

The change of the mixed-mode ratio is illustrated in Figure 5, where the mode ratio determined at point A can possibly change to a different value when the loading equilibrium moves to point B at the next time step upon a displacement jump, for instance. According to Pinho et al. [66], once the mode ratio changes, the damage history and the strategy to restore the cohesive energy are not clear. A solution was also proposed, where they resorted to the maximum mixed-mode displacement and employed a circle propagation criterion for the mixed-mode propagation criterion, regardless of the mode ratio.

This idea was adopted as the present initiation criterion. When associated with the non-dimensional scalar ψ, Equation (31) is rewritten as

(32) $$\psi =\sqrt{{\left(\frac{\delta_{n}}{\delta_{0}}\right)}^{2}+{\left(\frac{\delta_{s}}{\delta_{0}}\right)}^{2}},$$

with $\delta_{0}=\min\left\{\delta_{n0},{\;\delta }_{s0}\right\}$. The maximum of  ψ is recorded as

(33) $$\psi^{*}=\underset{0\leq t^{\prime }<t}{\max}\left\{\psi \left(t^{\prime }\right),\;\psi \left(t\right)\right\}.$$

When $\psi^{*}=1$, fracture initiates. Furthermore, two separation components can then be used to calculate the critical value $\lambda_{\mathrm{cr}}$ along with Equation (14). Note that this strategy in recording the maximum value to indicate fracture initiation was also employed in Reference [55].

Therefore, a circle initiation criterion is developed, as illustrated in Figure 5. It should be mentioned that this initiation criterion is similar to the propagation criterion [66] in the sense that both opening and shear modes can be activated simultaneously, which is more consistent with the fracture mechanism. Additionally, this treatment takes into account the concern of safe design, which was employed by Balzani and Wagner [61] for a propagation criterion. Furthermore, this initiation criterion essentially indicates the onset of the fracture process, and it plays a similar role to that used in extrinsic cohesive zone models [31,32,69].

To sum up, before $\psi^{*}$ reaches unity, the reversible connective model works; after satisfying this initiation criterion, the irreversible cohesive model comes into effect.

## 4. Contact Model: Representation of Particulate Materials

Once decohesion in a particle pair is completed, these two particles become in contact. The contact force is calculated based on the Hertz contact theory [70,71,72], which describes the relation between the contact force and the penetration between two particles of a pair.

**Figure 6:**
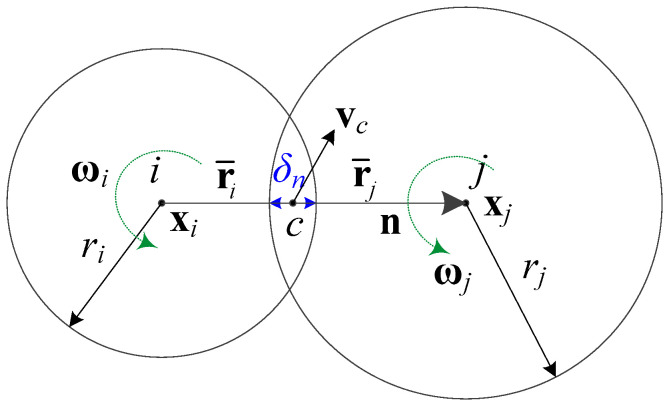
Hertz contact model based on two spherical particles.

When contact occurs between the two particles i and j, as shown in Figure 6, the Hertz contact model is used to calculate the contact force on particle i along the normal direction as

(34) $$f_{i\;\left(n\right)}^{\mathrm{con}}=-\frac{4}{3}\kappa \tilde{E}{\tilde{r}}^{1/2}\delta_{n}^{3/2}n,$$

where n denotes a unit normal vector, κ denotes a user-defined penalty factor, and $\tilde{E}$ is an equivalent Young’s modulus, as determined by

(35) $$\tilde{E}={\left(\frac{1-\nu_{i}^{2}}{E_{i}}+\frac{1-\nu_{j}^{2}}{E_{j}}\right)}^{-1},$$

where $\nu_{i}$ and $\nu_{j}$ are the Poisson’s ratios of the two contacting particles, and $\tilde{r}$ is the equivalent radius, given by

(36) $$\tilde{r}={\left(\frac{1}{r_{i}}+\frac{1}{r_{j}}\right)}^{-1}.$$

Lastly, $\delta_{n}$ is the penetration depth as determined by

(37) $$\delta_{n}=r_{i}+r_{j}-d,$$

where the distance d between the centers of two particles can be calculated with the use of two position vectors as

(38) $$d=\left(x_{j}-x_{i}\right)\cdot n.$$

When contact is persistent, Mindlin’s theory [71,73], which is based on the assumption that there is no partial slip between contacting particles, is used to calculate the tangential contact force on particle i as

(39) $$f_{i\;\left(s\right)}^{\mathrm{con}}=\frac{16}{3}\tilde{G}{\tilde{r}}^{1/2}\delta_{n}^{1/2}\delta_{s},$$

where $\tilde{G}$ is the equivalent shear modulus as determined by

(40) $$\tilde{G}={\left(\frac{2-\nu_{i}}{G_{i}}+\frac{2-\nu_{j}}{G_{j}}\right)}^{-1}.$$

G is the shear modulus, determined as G=E/2(1+ν), while $\delta_{s}$ denotes relative tangential displacement during the period $\tau^{\mathrm{con}}$ of persistent contact, expressed as

(41) $$\delta_{s}=\int_{\tau^{\mathrm{con}}}^{}v_{c\left(s\right)}\;dt,$$

where relative tangential velocity $v_{c\left(s\right)}$ at the contact point c is associated with the relative velocity $v_{c}$, determined by

(42) $$v_{c\left(s\right)}=v_{c}-\left(v_{c}\cdot n\right)n.$$

The relative velocity $v_{c}$ at the contact point c can be determined as

(43) $$v_{c}=v_{cj}-v_{ci},$$

where

(44) $$v_{ci}=v_{i}+\omega_{i}\times {\bar{r}}_{i},$$

(45) $$v_{cj}=v_{j}+\omega_{j}\times {\bar{r}}_{j},$$

where $v_{i}$ and $v_{j}$ are translational velocities of the particles, $\omega_{i}$ and $\omega_{j}$ are angular velocities of the particles, and ${\bar{r}}_{i}$ and ${\bar{r}}_{j}$ are effective radius vectors of the particles, as shown in Figure 6.

## 5. Model Transition: Monotonically from Connection to Contact

**Figure 7:**
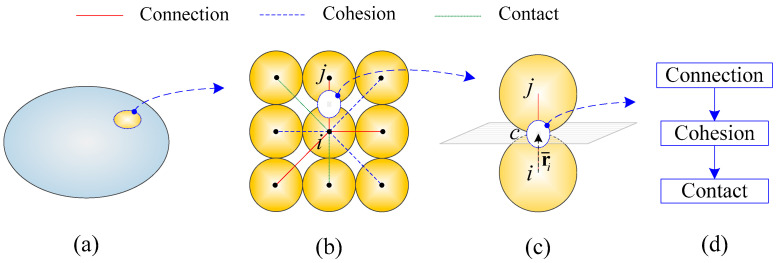
Model transition from a connective relation to cohesion and further to a contact relation: (**a**) an elastic solid represented by the discrete element connective model at microscale; (**b**) various relations among different particle pairs; (**c**) a representative particle pair; (**d**) monotonic model transition.

As fracture initiates and propagates, the model can transition from a connective model to a cohesive model and further to a contact model. The DE connective model, representing elastic solids at microscale, indicates the phase of a continuum. The contact model represents particulate materials. The particle-based CZM bridges the gap between them and describes the transition process of materials. The three models and the transition in between are illustrated in Figure 7. As the fracture process is irreversible, the model transition is monotonic toward the contact model. This monotonic model transition is consistent with the material transition.

Specific transition procedures are shown in Table 1. Initially, all particle pairs are in a connective relation and are employed to represent solids. The scalar ψ in the fracture initiation criterion of Equation (32) is used as the first transition criterion, and, once $\psi^{*}\geq 1.0$, the DE model changes its phase and becomes a cohesive model. Similarly, the scalar λ in the fracture propagation criterion of Equation (14) is used as the second transition criterion, and, once $\lambda^{*}\geq 1.0$, the DE particles become in contact condition.

It should be mentioned that, as shown in Figure 7b, a particle might be in different interaction manners with different surrounding particles in the meantime, while a particle pair, e.g., particle i and particle j, can only exhibit one certain interaction behavior, whether connective, cohesive, or contact.

## 6. Implementation: Explicit Update of Kinematics

The equations of motion of DE particles are expressed as

(46) $$m_{i}{\dot{v}}_{i}=f_{i}^{\mathrm{ext}}+f_{i}^{\mathrm{int}}+f_{i}^{\mathrm{coh}}+f_{i}^{\mathrm{con}},$$

(47) $$I_{i}{\dot{\omega }}_{i}=m_{i}+\sum_{j=1}^{N_{i}}{\bar{r}}_{ij}\times f_{ij},$$

where m and I are the particle mass and the moment of inertia, respectively, v and ω are translational and rotational velocities, respectively, and $\dot{v}$ and $\dot{\omega }$ are their first derivatives with respect to time; $f^{\mathrm{ext}}$ denotes external force and may include the contribution of coupling force if this particle is in a coupling interaction with the FE domain, and m denotes the moment induced from $f^{\mathrm{ext}}$; $f^{\mathrm{int}}$, $f^{\mathrm{coh}}$, and $f^{\mathrm{con}}$ are the internal force, cohesive force, and contact force contributed by the above-mentioned DE connective model, cohesive model, and contact model, respectively; $N_{i}$ is the number of surrounding particles around particle i; $\bar{r}$ denotes an effective radius and, in Figure 7c, it is a vector from the center of particle i to point c, which is located at the common plane between particle i and particle j; lastly, f is $f^{\mathrm{int}}$, $f^{\mathrm{coh}}$, or $f^{\mathrm{con}}$ because a specific particle pair can be in connection, in cohesion, or in contact.

The calculations of $f^{\mathrm{int}}$, $f^{\mathrm{coh}}$, or $f^{\mathrm{con}}$ are the main concern here, and the specific procedures are briefly summarized in Table 2. Note that tractions can be transformed to forces.

The central difference method was adopted here for explicit time integration. Since the central difference method is conditionally stable, the selected time step should be less than the critical one. For the selection strategy of the time step, the reader can refer to [74] for detail. Velocities and relative displacement are assumed to be known at time $t_{\left(n\right)}$. At any time, the translational acceleration and rotational acceleration can be calculated from Equations (46) and (47), respectively. Supposing the incremental time step from time $t_{\left(n\right)}$ to $t_{\left(n+1\right)}$ is Δt, the translational and rotational velocities at the mid-point of that period, i.e., $t_{\left(n+1/2\right)}$, can be respectively calculated by

(48) $$v_{\left(n+1/2\right)}=v_{\left(n\right)}+\frac{1}{2}\Delta t{\dot{v}}_{\left(n\right)},$$

(49) $$\omega_{\left(n+1/2\right)}=\omega_{\left(n\right)}+\frac{1}{2}\Delta t{\dot{\omega }}_{\left(n\right)}.$$

The relative velocity ${\bar{v}}_{c}$ at the contact point c, which is on the common plane (see Figure 7c), between a particle pair (i and j) can, therefore, be calculated as

(50) $${\bar{{\bar{v}}_{c}}}_{\left(n+1/2\right)}=v_{cj\left(n+1/2\right)}-v_{ci\left(n+1/2\right)},$$

where

(51) $$v_{cj\left(n+1/2\right)}=v_{j\left(n+1/2\right)}+\omega_{j\left(n+1/2\right)}\times {\bar{\bar{r}}}_{j\left(n\right)},$$

(52) $$v_{ci\left(n+1/2\right)}=v_{i\left(n+1/2\right)}+\omega_{i\left(n+1/2\right)}\times {\bar{\bar{r}}}_{i\left(n\right)}.$$

Then, the incremental relative displacement vector $\Delta \delta_{\left(n+1\right)}$ from time $t_{\left(n\right)}$ to $t_{\left(n+1\right)}$ can be updated by

(53) $$\Delta \delta_{\left(n+1\right)}=\Delta t{\bar{v}}_{c}{}_{\left(n+1/2\right)},$$

and the total relative displacement vector $\delta_{\left(n+1\right)}$ at time $t_{\left(n+1\right)}$ is updated as

(54) $$\delta_{\left(n+1\right)}=\delta_{\left(n\right)}+\Delta \delta_{\left(n+1\right)},$$

which is essential for the calculation of internal, cohesive, and contact forces.

## 7. Numerical Simulations

The particle-based cohesive crack model was validated using three numerical simulations of standard fracture tests and applied to the impact fracture of a notched concrete beam. These standard fracture tests included a double cantilever beam (DCB) model, an end notched flexural (ENF) model, and a mixed-mode bending (MMB) model. These three fracture tests correspond to mode-I (opening mode), mode-II (shear mode), and a mixed mode between I and II, respectively.

It is assumed that the material’s macroscopic behavior is determined by the material’s micro-structure and the interplay between particles at its associated scale. Therefore, the proposed microscopic cohesive crack model can be validated using the macro-structural loading–deflection relation of these fracture tests, which can be analytically determined based on the linear elastic fracture mechanics (LEFM). For these three models, cracks occur only on their mid-plane and, thus, cohesive elements are pre-defined as a weak layer.

The primary material properties for the isotropic solids were adopted from References [62,75], where E= 120 GPa, $G_{Ic}=$ 0.26 N/mm for the opening mode, and $G_{\mathrm{II}c}=$ 1.002 N/mm for the shear mode. In addition, density  ρ= 2500 kg/m^3^, Poisson’s ratio  ν= 0.2, and cohesive strength $\sigma_{c}=$ 70 MPa. The DE particle radius was set to r= 0.125 mm.

In the numerical simulations, loading was in displacement control with a velocity prescribed at selected DE particles. The prescribed velocity v went up to a certain value $v_{0}$ within a duration $t_{0}$, as expressed in the following form:

(55) $$v=\{\begin{array}{c} v_{0}t/t_{0}\;\;\;(t<t_{0})\\ v_{0}\;\;\;\;\;\;\;\;\;\;\;\;\left(t\geq t_{0}\right) \end{array}.$$

Note that $v_{0}=$ 0.075 m/s and $t_{0}=$ 0.5 ms apply to the numerical simulations of DCB, ENF, and MMB models; otherwise, an explicit statement would be made. It also should be mentioned that these simulations are for the quasi-static loading condition. Hence, the dynamic relaxation strategy via global damping is employed to make sure that the kinetic energy is less than 0.001% of the total energy [68].

The definition of the critical energy release rate on the DE model needs to be modified because of its special spatial structure. According to Irwin’s energy approach for fracture [76], the energy release rate is a measure of the energy available for an increment of crack extension. On the basis of the linear elastic fracture mechanics (LEFM), this energy release rate is also equal to its critical value $G_{c}$. Therefore, for the present beam analysis with a crack width B and length a, the critical energy release rate can be determined in association with the potential  Π of the beam as follows [9]:

(56) $$G_{c}=-\frac{1}{B}\frac{\partial \Pi }{\partial a}.$$

Note that the potential is related to the strain energy and the external work on the beam, and the kinematic energy is ignored for the quasi-static loading condition [9].

Considering the geometry of the present DE model, as shown in Figure 1b, it is found that the boundary DE particles in the model only have a connection with bulk particles and lose half their interaction on the other side. Thus, the width B needs to be corrected to B−2r when used in Equation (49). This effect, however, diminishes with the increase of width.

### 7.1. Mode-I Validation: Numerical Analysis of a DCB Model

**Figure 8:**
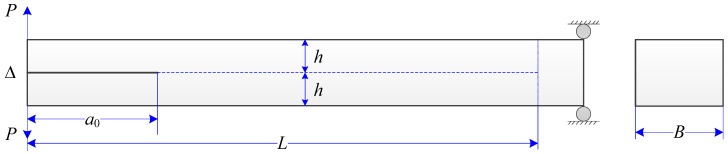
Configuration, loading, and boundary condition of a double cantilever beam (DCB) model.

The DCB model shown in Figure 8 is widely used to test fracture behavior in opening mode. The dimensions of the model were as follows: length L=30 mm, breadth B=2 mm, height 2h=2 mm, and initial crack length $a_{0}=9$ mm. According to the beam theory, the general form of the beam load–deflection relation is given as

(57) $$\Delta =\frac{2P{\left(a+\chi_{I}h\right)}^{3}}{3E𝓘},$$

where E is the Young’s modulus, $𝓘={\mathrm{Bh}}^{3}/12$ is the second moment of area of one arm of the cantilever, and $\chi_{I}$ is a mode-I correction factor for calculating an effective crack length to account for the root rotation and the shear deformation of the beam [77,78,79]. For isotropic solids used in the present study, this correction factor $\chi_{I}$ associated with mode-I fracture is estimated as follows [77]:

(58) $$\chi_{I}=\sqrt{\frac{E}{11G}\left(3-2{\left(\frac{\Gamma }{1+\Gamma }\right)}^{2}\right)},$$

where shear modulus  G=E/2(1+ν), and Γ is the elastic modulus correction parameter, which is given by

(59) Γ=1.18E/G.

**Figure 9:**
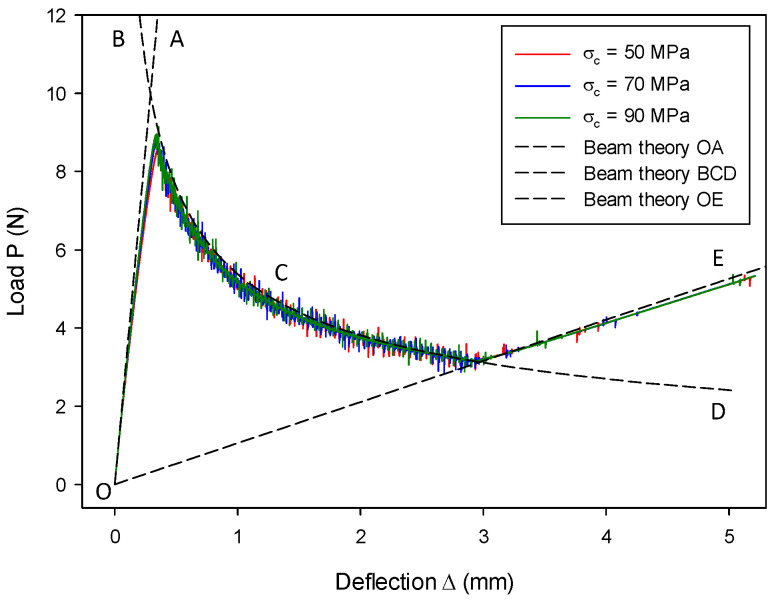
End load P as a function of deflection Δ in the DCB model with different cohesive strengths.

The initial loading line OA (stage-I) in Figure 9 corresponds to a stationary crack with an initial crack length $\;a=a_{0}$ and, thus, Equation (57) can be rewritten as

(60) $$\Delta =\frac{2P{\left(a_{0}+\chi_{I}h\right)}^{3}}{3E𝓘}.$$

When the crack propagates beyond the initial crack length, i.e., $a>a_{0}$, the crack length is associated with the total energy release rate $G_{T}$. For the fracture problem of mode-I, $G_{T}=G_{\mathrm{Ic}}$. Therefore, the crack length can be obtained by $\;a=\sqrt{\left(B-2r\right)EG_{\mathrm{Ic}}}/P-\chi_{I}h$ based on the corrected beam theory [80]. Then, the line BCD (stage-II) in Figure 9 can be described by

(61) $$\Delta =\frac{2P{\left(\left(B-2r\right)E𝓘G_{Ic}\right)}^{3/2}}{3E𝓘P}.$$

After the crack propagates to the end of the initial weak layer, i.e., a=L, each cantilever beam behaves independently. Therefore, the line OE (stage-III) in Figure 9 is described by the following equation according to the beam theory:

(62) $$\Delta =\frac{2P{\left(L+\chi_{I}h\right)}^{3}}{3E𝓘}.$$

**Figure 10:**
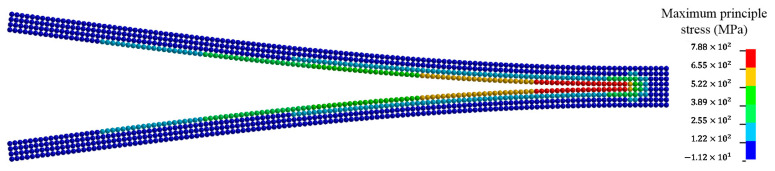
Deformed configuration of the DCB model at t=35 ms.

In numerical simulations of the DCB model where $G_{c}=G_{\mathrm{Ic}}$, loads are in displacement control along both upward and downward directions, as seen in Figure 8. Load is applied to both top and bottom DE particles on the left side. After crack propagation is completed, the final configuration can be seen from Figure 10.

The numerical results agree with the analytical solution at all three stages, as can be seen from Figure 9. Slight noise is observed since the fracture model was implemented in an explicit time-integration code. Different cohesive strengths were employed to investigate the load–deflection behavior as shown in Figure 9, where the difference was generally indistinguishable except for the peak loads. It is understood from Equation (18) that, for a given $G_{c}$, the size of the cohesive zone decreases with increasing cohesive strength, which results in materials tending to be more brittle. As a result, numerical results can better match the LEFM-based analytical solution.

**Figure 11:**
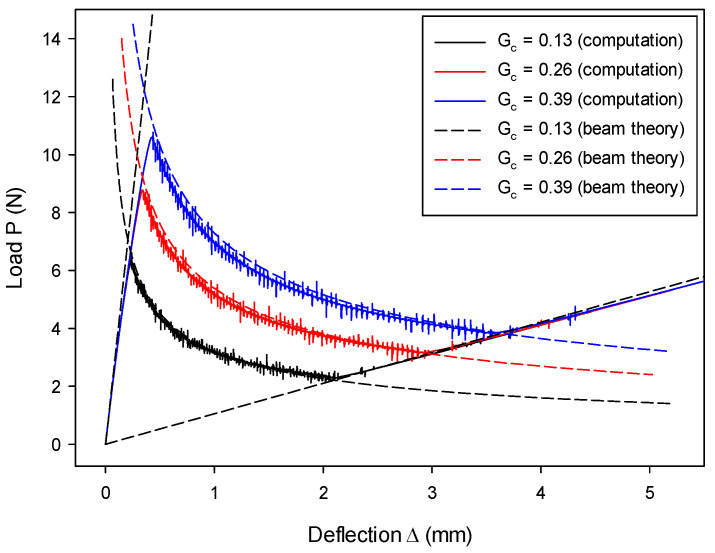
Load–deflection (P~Δ) histories vary as a function of critical energy release rate $G_{Ic}$.

The influence of the critical energy release rate $G_{\mathrm{Ic}}$ on the load–deflection relation was investigated, and the results are shown in Figure 11. Numerical results computed from the proposed model are in good agreement with the analytical solution in all three stages for all three cases, i.e., $G_{\mathrm{Ic}}=$ 0.13 N/mm^2^, 0.26 N/mm^2^, and 0.39 N/mm^2^. With the increase of $G_{Ic}$, only the line in the second stage changes, as determined by Equation (54). Since the initial loading stage and the complete split stage are with a stationary crack, the load–deflection relation at these two stages is irrelevant to $G_{\mathrm{Ic}}$. This is predicted by Equations (53) and (55) and confirmed by Figure 11.

**Figure 12:**
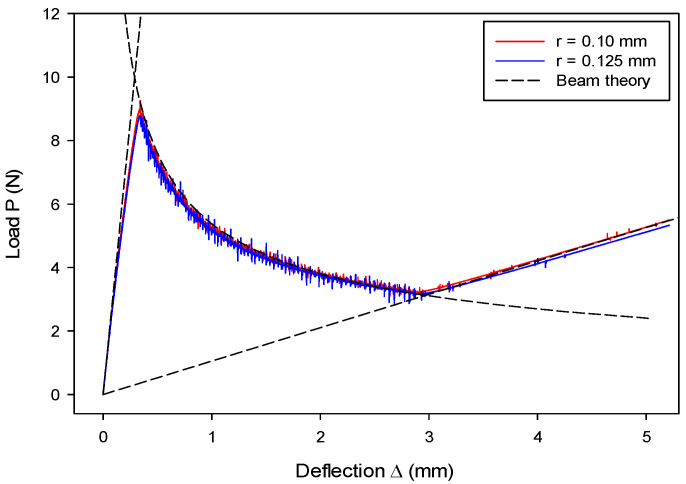
Size effect due to varying particle radius on the DCB model.

**Figure 13:**
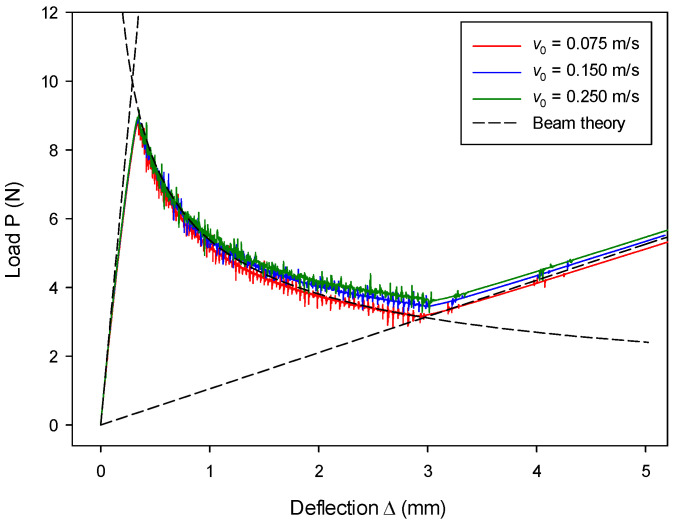
Loading rate effect on load–deflection relation.

The size effect from different DE particle radii was examined, and results are shown in Figure 12. The results with r= 0.10 mm match the results with r= 0.125 mm, as well as the analytical results. This indicates that the size effect is insignificant. The loading rate’s effect on the load–deflection (P~Δ) relation is shown in Figure 13. The numerical results are in good agreement with the analytical solution, but they slightly deviate at the later second stage. It is also found that a larger loading rate leads to a larger discrepancy. The discrepancy is probably due to the fact that, when a larger loading rate is adopted in an explicit time-integration code, a larger inertia effect should be accounted for, which is against the quasi-static assumption of the LEFM-based analytical solution.

**Figure 14:**
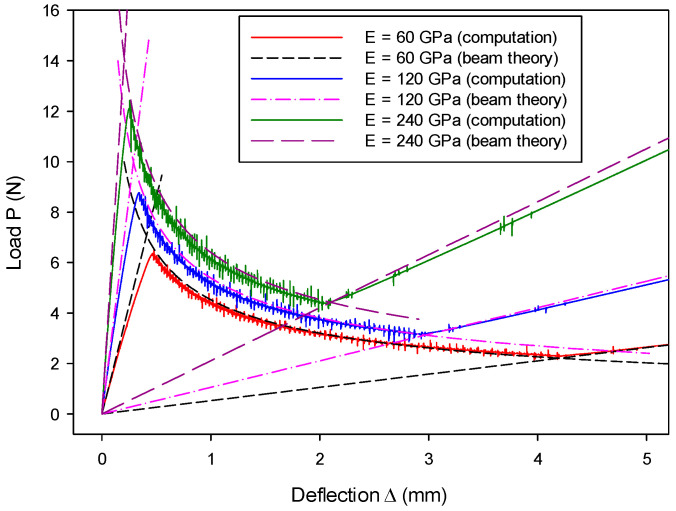
Young’s modulus effect on load–deflection relation.

The CZM was constructed at the particle bonding springs. It is found from Equation (3) that the micromechanical parameters of spring stiffness $k_{n}$ and $k_{s}$ are correlated to the macroscopic material parameters of Young’s modulus E and Poisson’s ratio  ν, apart from particle radius r. Hence, the effect of the variation of E and ν was also examined. The effect of Young’s modulus E on the load–deflection relation is shown in Figure 14, where it can be seen that numerical results computed from the proposed model agree well with the analytical solutions for all three stages. It can be seen from Equation (3) that the increase of macroscopic E results in an increase of the micro-structural spring stiffness. The change of micromechanical parameters leads to varying macroscopic performance, albeit consistent with the respective analytical predictions. Moreover, Figure 14 shows a greater E leading to a higher peak value and a shorter fracture process, which is consistent with the prediction by Equations (60)–(62).

**Figure 15:**
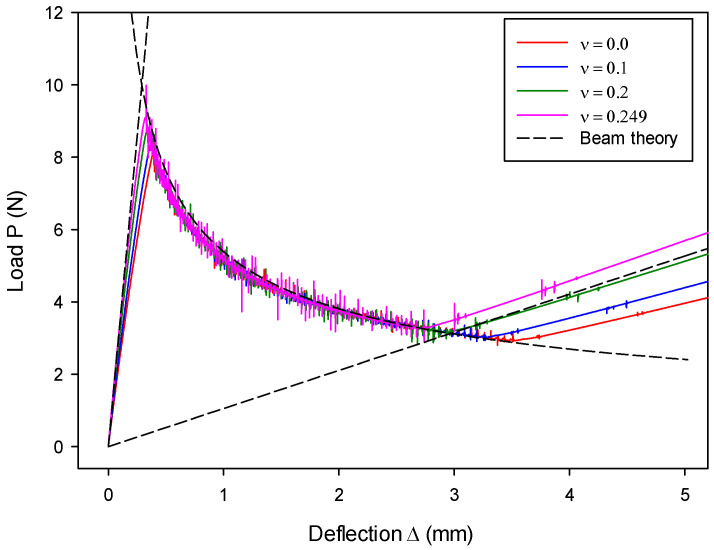
Poisson’s ratio effect on load–deflection relation.

The effect of Poisson’s ratio ν on the load–deflection relation is shown in Figure 15. The computed results with various values of ν are generally in good agreement with the analytical solution, particularly the results on fracture propagation in stage-II. For stage-I and stage-III, it is observed that a smaller magnitude of ν leads to a slightly later start of the fracture process, as well as a later closure. This behavior indicates that the beam with a smaller ν possesses a slightly lower macro-structural bending stiffness. The discrepancies in stage-I and stage-III are due to the spring stiffness. The stiffness of springs changes with the variation of ν and, thus, it leads to a slightly different macro-structural response on the current beam, which uses a limited number of DE particles (4096 particles on each beam). Nonetheless, this discrepancy may be reduced with the increasing number of DE particles used in the beam.

### 7.2. Mode-II Validation: Numerical Analysis of an ENF Model

**Figure 16:**
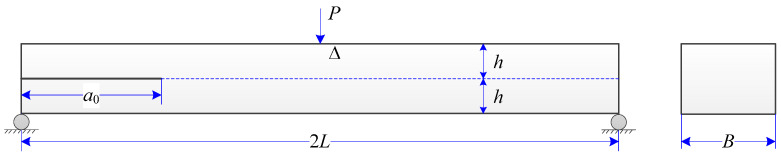
Configuration, loading, and boundary condition of an end notched flexural (ENF) model.

**Figure 17:**
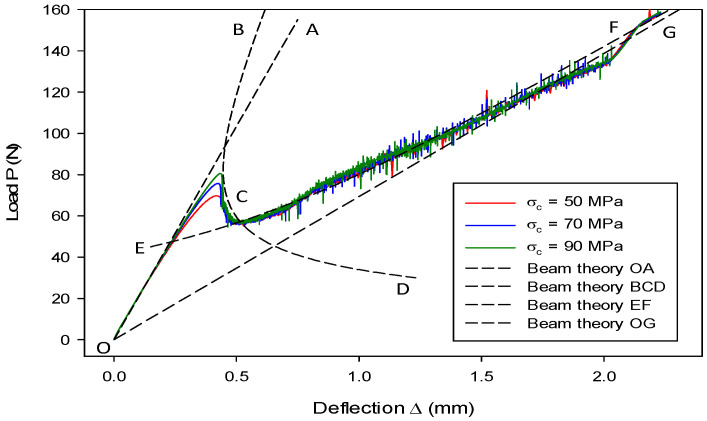
Load P as a function of deflection Δ in the ENF model with different cohesive strengths.

The ENF model, as shown in Figure 16, is widely used to simulate fracture processes under shear-mode loading. The dimensions of the model were as follows: length 2L=30.25 mm, breadth B=2 mm, height 2h=2 mm, and initial crack length $a_{0}=9$ mm. In the context of simple beam theory [57,81], the analytical solutions for the line OA and the line BCD in Figure 17 take the same form. When considering the corrected beam theory [78], the analytical form can be written as

(63) $$\Delta =\frac{P{\left(2L^{3}+3\left(a+\chi_{II}h\right)\right)}^{3}}{3E𝓘},$$

where $\chi_{\mathrm{II}}$ is a correction factor similar to $\chi_{I}$, associated with mode-II fracture. This factor only takes into account the shear factor and does not include root rotation [82]; hence, its value is less than that used for the DCB model. It is estimated to be $\chi_{\mathrm{II}}=0.42\chi_{I}$ [82].

For the initial loading line OA (stage-I) in Figure 17, the crack is stationary and $a=a_{0}$. Therefore, the analytical solution of the load–deflection relation can be obtained straightforwardly after substituting $a_{0}$ into Equation (63). By contrast, the crack length a for the line BCD in Figure 17 is related to the critical energy release rate $G_{\mathrm{IIc}}$, i.e., $a=\sqrt{64\left(B-2r\right)EG_{\mathrm{IIc}}/3P^{2}}-\chi_{\mathrm{II}}h$ [57]. When substituting it into Equation (63), the analytical solution of the line BCD (stage-II) is obtained. Note that, at this stage, the crack length is limited to the mid-span, i.e., a<L.

When the crack propagates beyond the mid-span, i.e., a>L, the analytical solution of the line EF (stage-III) in Figure 17 is given by Equation (64) [81].

(64) $$\Delta =\frac{P\left(8L^{3}-3{\left(2L-\left(a+\chi_{II}h\right)\right)}^{3}\right)}{96E𝓘},$$

where $\;a=2L-\sqrt{64\left(B-2r\right)EG_{\mathrm{IIc}}/3P^{2}}-\chi_{\mathrm{II}}h$.

Once the beams are completely split, the load–deflection relation of the line OG (stage-IV) is simply given by Equation (65) [57].

(65) $$\Delta =\frac{PL^{3}}{12E𝓘}.$$

**Figure 18:**
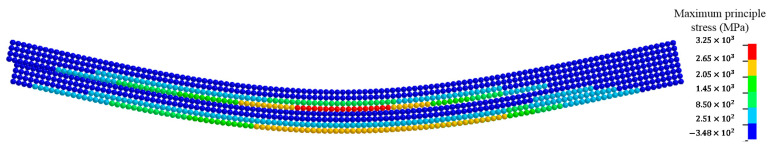
Deformed configuration of the ENF model at t=30 ms.

In numerical simulations of the ENF model where $G_{c}=G_{\mathrm{IIc}}$, load P is applied at the mid-span, and deflection Δ is also measured there. The deformed configuration is shown in Figure 18. The overall performance agrees very well with the analytical prediction. The crack propagation is completed after the deflection of the mid-span Δ exceeds 2.0 mm. Then, the two beams deform as an entity, and the load–deflection relation matches the beam theory, which is described by the line OG. Different cohesive strengths are used, and the difference can only be observed from the transition between stage-I and stage-II. It is not surprising to see that a higher cohesive strength results in a better agreement since materials behave in a more brittle way. Additionally, it is worth noting that, once cracks start initiate, the fracture behavior associated with the present particle-based CZM is generally insensitive to the cohesive strength variation, which was also observed by Harper and Hallet [75].

**Figure 19:**
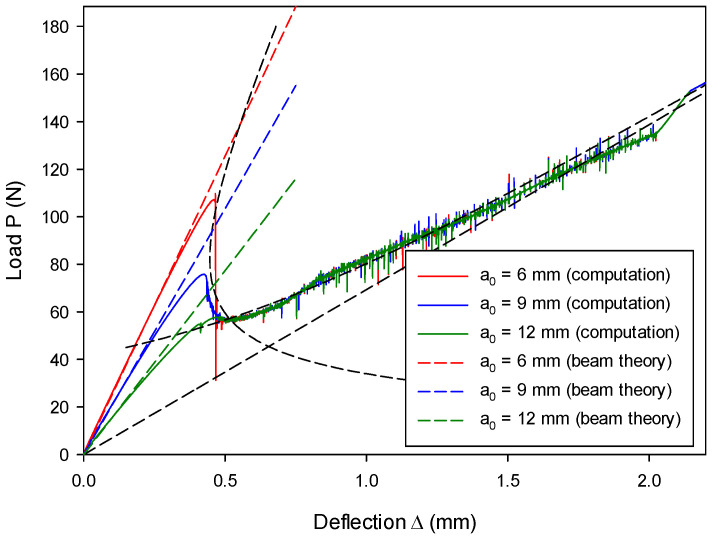
Load–deflection (P~Δ) histories vary as a function of initial crack lengths $a_{0}$.

The influence of initial crack length on the load–deflection relation can be seen from Figure 19. Various initial crack lengths ($a_{0}=$ 6 mm, 9 mm, and 12 mm) were adopted and, overall, the computational results from the proposed model are in good agreement with the analytical ones. Obviously, with a shorter initial crack length, the beam is of a higher bending stiffness and, thus, the initial loading tends to be more resistant. This is consistent with the beam theory. It should be mentioned that, if the initial crack is too short ($a_{0}=$ 6 mm in this case), the instability phenomenon of snap-back occurs (see Figure 19). Because the present loading is in a displacement control, the mid-span deflection would monotonically increase, which violates the analytical prediction that deflection can decrease, as shown in Figure 19. This dynamic snap-back was also postulated to exist in reality by Mi et al. [57], consistent with the experimental observation [83] that instability takes place when ENF tests are conducted with an insufficient initial crack length. It should also be noted that, even though snap-back occurs, the remaining load–deflection relation still follows the correct path until both beams completely split.

**Figure 20:**
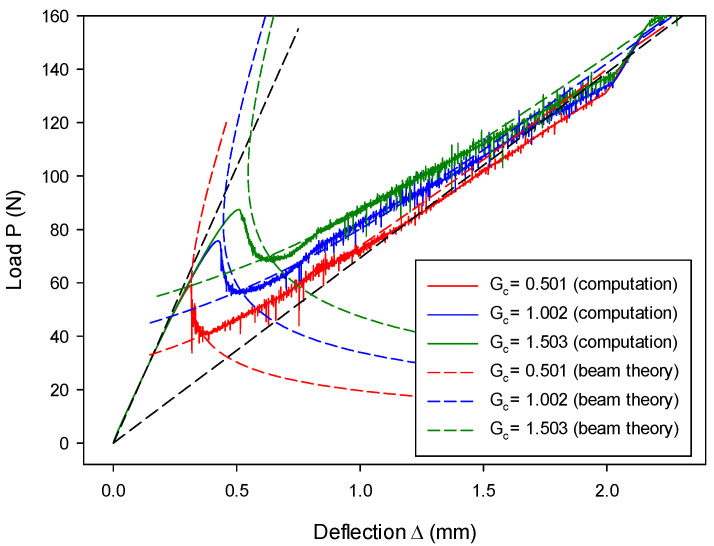
Load–deflection (P~Δ) histories vary as a function of critical energy release rate $G_{c}$.

The influence of the critical energy release rate on the load–deflection relation can be seen from Figure 20, where results in stage-II and stage-III are affected but remain almost unaffected in stage-I and stage-IV. This observation is in agreement with the analytical prediction, as seen in Figure 20 and the theory of Equations (63) and (64), since only the crack length a is affected. Furthermore, it is confirmed that the critical energy release rate plays a significant role in fracture behavior.

### 7.3. Mixed-Mode I & II Validation: Numerical Analysis of an MMB Model

**Figure 21:**
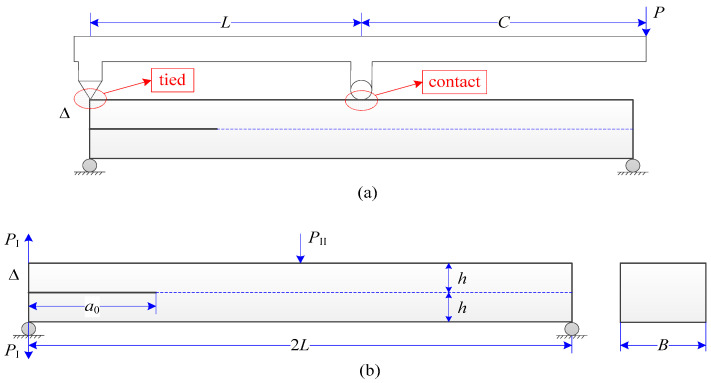
Configuration, loading, and boundary condition of a mixed-mode bending (MMB) model: (**a**) experimental set-up; (**b**) numerical set-up.

The MMB test (shown in Figure 21) was designed by Reeder and Crew [84] to provide a wide range of mixed-mode ratios by adjusting the length of the loading arm. The dimensions of the model were as follows: length 2L=30.25 mm, breadth B=2 mm, height 2h=2 mm, and initial crack length $a_{0}=9$ mm. An MMB model is equivalent to the superposition of a DCB model and an ENF model. Therefore, the analytical solution of the MMB model can be attained via superposition. Given the loading P and the loading arm C, as shown in Figure 21, the pure mode-I loading $P_{I}$ and mode-II loading $P_{\mathrm{II}}$ can be determined by Equation (66) [84].

(66) $$P_{I}=\frac{\left(3C-L\right)}{4L}P;\;\;\;P_{II}=\frac{\left(C+L\right)}{L}P.$$

The mixed-mode ratio $\;\beta =G_{\mathrm{II}}/G_{T}$, where $G_{T}=G_{I}+G_{\mathrm{II}}$ and $G_{I}$ and $G_{\mathrm{II}}$ are energy release rates corresponding to the opening mode and shear mode, respectively. This ratio changes with the adjustment of the loading arm, and the specific relation is expressed by Equation (67) [84].

(67) $$C=\frac{\left(1+\frac{1}{2}\sqrt{\frac{3\left(1-\beta \right)}{\beta }}\right)}{\left(3-\frac{1}{2}\sqrt{\frac{3\left(1-\beta \right)}{\beta }}\right)}.$$

The  P~Δ relation, as seen in Figure 21, is only decided by the DCB model component [81], and it can be specifically given by

(68) $$\Delta =\frac{2P_{I}{\left(a+\chi_{I}h\right)}^{3}}{3E𝓘}=\frac{\left(3C-L\right){\left(a+\chi_{I}h\right)}^{3}}{6E𝓘L}P.$$

In the initial loading with a stationary crack $a_{0}$, the analytical solution of the line OA (stage-I)23, can be obtained by replacing a by $a_{0}$ in Equation (68).

When the crack occurs and proceeds, but with a crack length less than L, the length of crack, a, can be determined by the following equation:

(69) $$\frac{P_{I}{}^{2}{\left(a+\chi_{I}h\right)}^{2}}{BE𝓘G_{Ic}}+\frac{3P_{II}{}^{2}{\left(a+\chi_{II}h\right)}^{2}}{64BE𝓘G_{IIc}}=1$$

Then, the  P~Δ relation of the line BCD (stage-II) can be obtained by substituting a into Equation (68).

When the crack propagates beyond the mid-span, i.e., a>L, the crack length can be determined by solving the following equation [57]:

(70) $$\frac{1}{8BE𝓘}\left(\frac{8P_{I}^{2}}{G_{Ic}}+\frac{3P_{II}^{2}}{8G_{IIc}}-\frac{8P_{I}P_{II}}{G_{IIc}}\right)a^{2}-\frac{L}{8BE𝓘}\left(\frac{3P_{II}^{2}}{2G_{IIc}}-\frac{8P_{I}P_{II}}{G_{IIc}}\right)a+\frac{3P_{II}^{2}L^{2}}{16BE𝓘G_{IIc}}=1.$$

Thus, the line EF (stage-III) can be obtained by substituting a into Equation (68).

**Figure 22:**
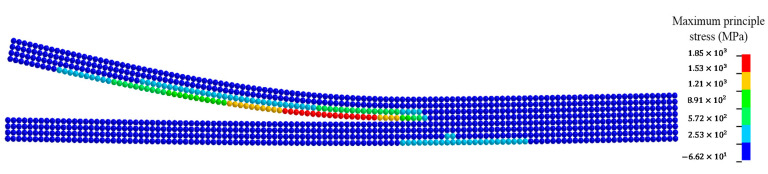
Deformed configuration of the MMB model (β= 0.5) at t= 30 ms.

The experimental set-up of an MMB model is schematically illustrated in Figure 21. The model is loaded by a lever with two loading points, where the end loading is in a tied condition and the middle one is a contact condition. Since the lever is considered as a rigid body, the load P and its displacement can be related to the loads and displacements at the left end and at the middle points. Their specific relations are given in Reference [60]. This strategy was also adopted in the present numerical model, where the lever was not simulated for simplicity (see a deformed configuration of the numerical model in Figure 22). The consequence of this numerical set-up is that loads at the left end and at the middle have to be tied with the model. Therefore, the loading condition at the middle is different from the contact condition used in the experimental set-up. This treatment is reasonable when the loading arm is not too short.

**Figure 23:**
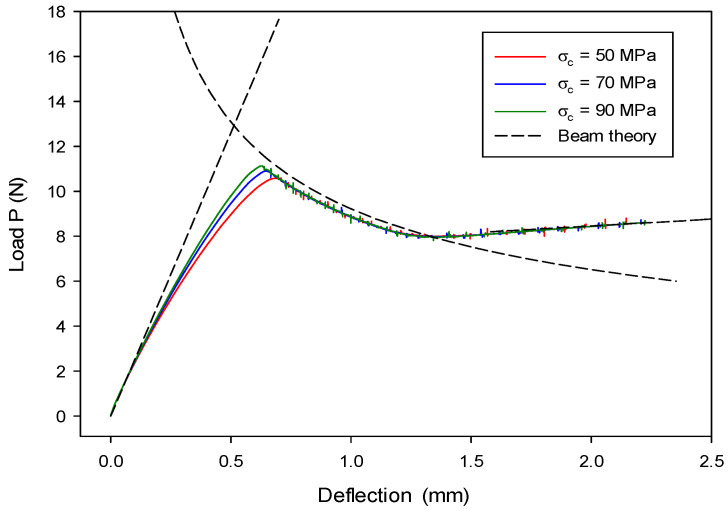
End load P as a function of deflection Δ in the MMB model (β= 20%) with different cohesive strengths.

**Figure 24:**
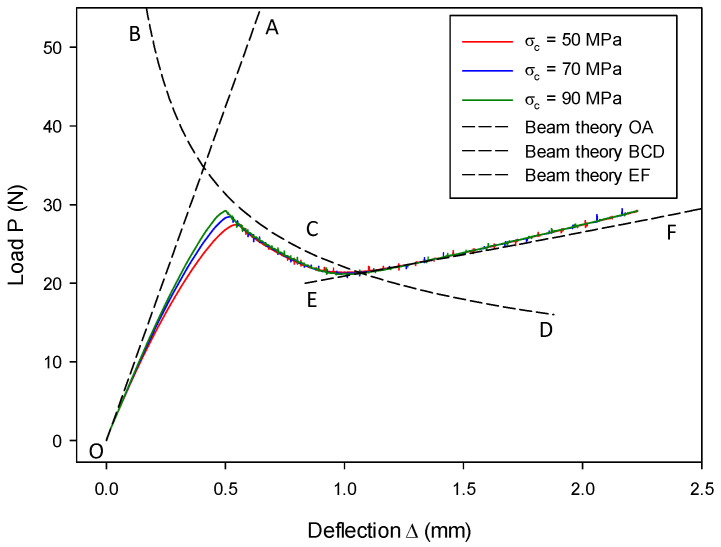
End load P as a function of deflection Δ in the MMB model (β= 50%) with different cohesive strengths.

**Figure 25:**
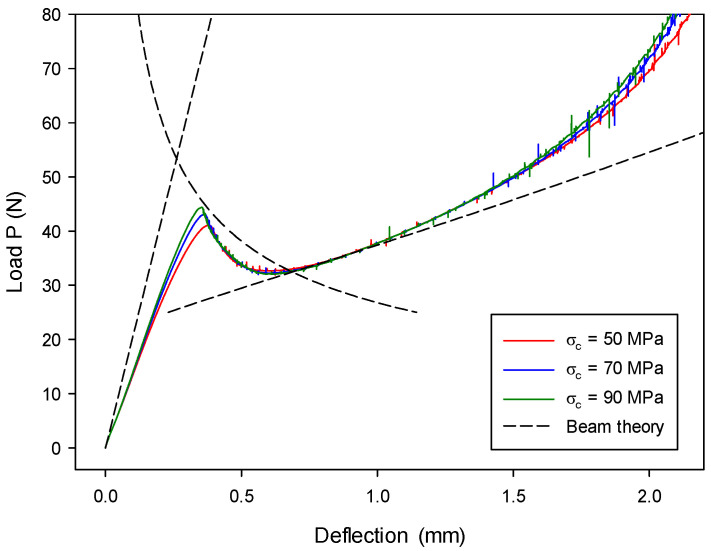
End load P as a function of deflection Δ in the MMB model (β= 80%) with different cohesive strengths.

Three different cases with different mixed-mode ratios, i.e., β= 20%, 50%, and 80%, were modeled, and different lengths of loading arm, i.e., C= 32.590 mm, 13.226 mm, and 8.443 mm, were set correspondingly. The computed results (with assuming $G_{c}=G_{\mathrm{IIc}}$) from the proposed model are shown in Figure 23, Figure 24 and Figure 25, and they are compared with the analytical solution mentioned above. When β= 20% and 50%, the computed results are in good agreement with the analytical solution in all three stages ($a=a_{0}$, a<L, and a>L). The computed results with β= 80%, as shown in Figure 25, overall agree with the analytical solution, particularly for the first two stages. However, they gradually deviate from the analytical results in stage-III with the increase of deflection. This discrepancy is caused by the approximate treatment of the loading condition at the middle, being tied instead of in contact. Specifically, when the loading arm C becomes smaller, the deflection at the mid-span tends to be larger for a certain deflection Δ at the left end. Therefore, the middle loading point has a bigger trend moving toward the left end because of the rotational effect between the left-end loading and the middle-point loading. The tied condition adopted in this research apparently resists this motion, but the rolling contact condition usually adopted in the experiment does not. It should also be mentioned that the trend of this left motion is pretty small in the models with β= 20% and 50%; thus, the tied loading condition at the middle is effective, and their performance in stage-III is close.

In each case with different mixed-mode ratio, the influence of cohesive strength on the load–deflection relation was investigated. As observed from Figure 23, Figure 24 and Figure 25, the influence on the fracture behavior (at lines BCD and EF) is overall insignificant, but computed results at the initial loading stage approach the analytical solution with a greater cohesive strength. This is because material tends to be more brittle with a higher cohesive strength, which better matches the assumption of the LEFM-based analytical solution.

**Figure 26:**
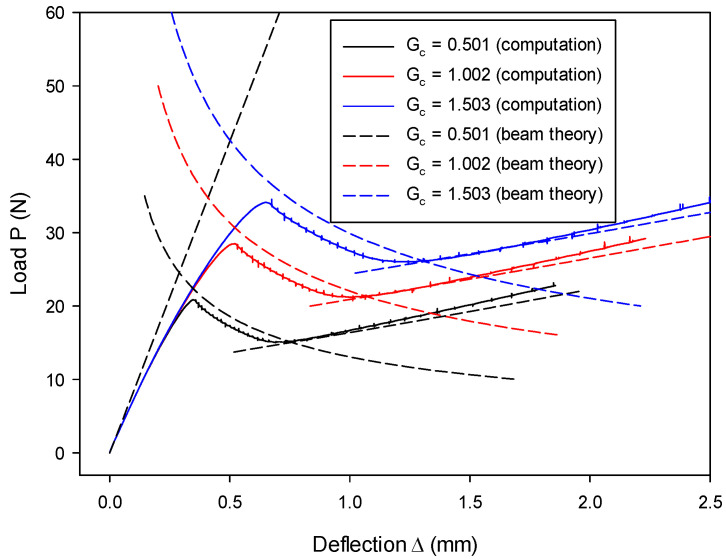
Load–deflection (P~Δ) histories in the MMB model (β= 50%) vary as a function of critical energy release rate $G_{c}$.

The effect of different fracture energies was also examined with β= 50%. It can be seen from Figure 26 that all three cases with different $G_{c}$ match their analytical results. Furthermore, varying fracture energy has an impact on crack propagation stages but not on the initial loading stage. This is because crack length a is different (see Equations (69) and (70)) as a result of varying $G_{c}$.

### 7.4. Application to the Impact Fracture of a Notched Concrete Beam

**Figure 27:**
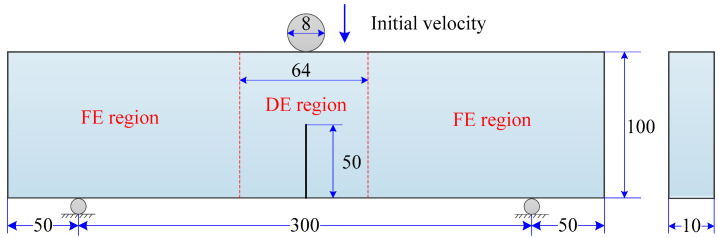
Geometry and loading condition of the concrete beam model.

Concrete is a quasi-brittle material, and concrete beams are often used to investigate the mechanical performance and the fracture behaviors of the material when subjected to dynamic loading conditions. A significant number of studies on this were carried out experimentally [85,86] and numerically using the FEM associated with the CZM [87] and with continuum damage mechanics [88]. Here, the dynamic fracture behavior of a plain concrete beam, which has an initial notch at the mid-span as shown in Figure 27, was studied using the proposed cohesive crack model. The same model was also studied experimentally by Zhang et al. [86] and studied numerically by Bede et al. [88] using the FEM associated with continuum damage mechanics.

The geometry and loading condition are depicted in Figure 27. The notched concrete beam was supported at its two ends and subjected to an impact at the mid-span. The length, width, and depth of the concrete beam were 400 mm, 10 mm, and 100 mm, respectively. Note that the width of the original geometric model in References [86,88] was 100 mm. Since this model is essentially a two-dimensional problem because there is no need to distinguish the difference between the front side and the back side, a 10% proportion was used instead to save computation time. An initial notch of height of 50 mm was located at the mid-span in the bottom of the beam. Two supporters were located 50 mm away from their respective edges of the beam. The impactor was a rigid cylinder featuring a diameter of 8 mm and a mass of 12.06 kg with an initial velocity of 1.76 m/s.

The concrete beam had a density of 2368 kg/m^3^, Young’s modulus of 31 GPa, Poisson’s ratio of 0.18, tensile strength of 5.4 MPa, and critical energy release rate of 141 N/m. The supports were regarded as elastic material with a density of 7900 kg/m^3^, Young’s modulus of 210 GPa, and Poisson’s ratio of 0.20. The Young’s modulus and Poisson’s ratio of the impactor were 210 GPa and 0, respectively.

**Figure 28:**
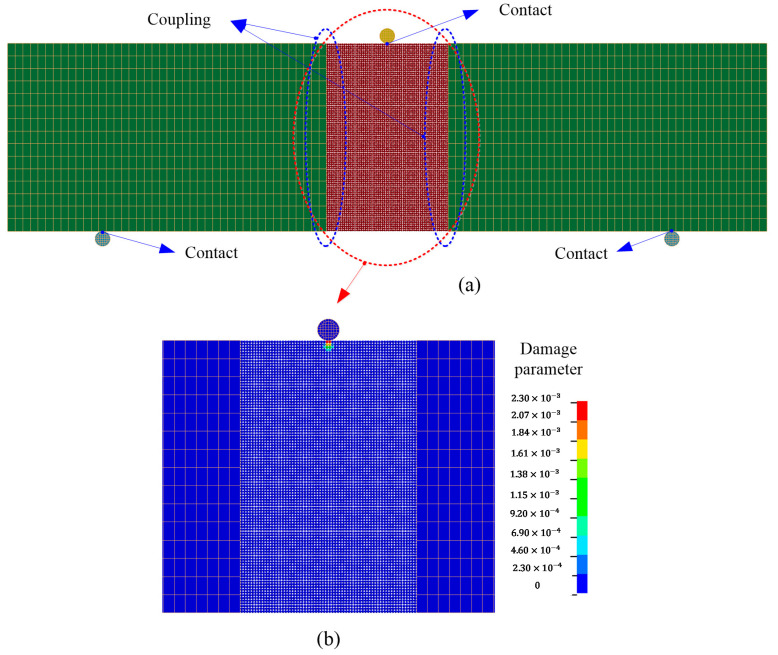
(**a**) Coupled model; (**b**) initial damage upon impact at t=0.04 ms.

The combined finite–discrete element method was used to model this impact problem. The concrete domain with a range of 64 mm just below the impactor was discretized by DEs to better characterize the fracture behavior, and FEs were used to discretize the two sides of the beam to save computation time. Different element patterns are shown in Figure 28a. The radius of DE particles was 0.5 mm, and there were a total of 64,000 DE particles and 6048 FE hexahedrons. The time step was automatically calculated to keep computation stable, as required by the adopted central difference method.

As the domain was composed of separate regions, the interaction between different regions was enforced by the coupling approach, as detailed in Reference [40]. The impactor and the two supporters were discretized by hexahedron FEs. The contact between the FE regions and the supports was of FE/FE contact type, while the contact between the impactor and the DE region was of FE/DE contact type. The possible coupling and contact interfaces are shown in Figure 28a. The DE region of the concrete beam initially employed the connective model to represent a continuum. Once this region was under certain deformation and fracture occurred, some DE particles changed their connecting state from connection to cohesion, and they could even be in contact. This model transition was simulated using the proposed cohesive crack model. All above-mentioned contact problems were addressed using the proposed unified contact algorithm, as detailed in Reference [40].

Upon the impact from the cylinder, damage appeared on the top surface of the DE region, as shown in Figure 28b. Note that the damage parameter was defined in Equation (22). This indicates that some DE particles changed their connecting state from the connective model to the cohesive models, but no full decohesion was observed before the damage initiated from the pre-set notch. Note that the occurrence of this damage was just because of the local failure due to an instantaneous impact.

**Figure 29:**
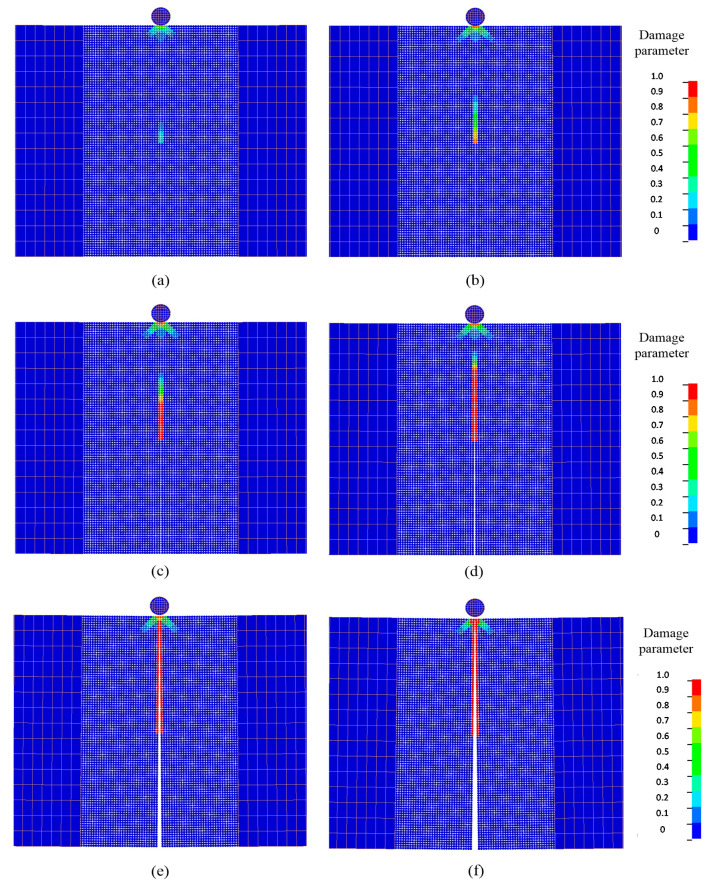
Evolutions of crack and damage in the concrete beam with an initial notch at the mid-span: (**a**) t=0.16 ms; (**b**) t=0.20 ms; (**c**) t=0.24 ms; (**d**) t=0.32 ms; (**e**) t=0.58 ms; (**f**) t=0.79 ms.

Typical fracture patterns and damage evolutions in the beam at different times are displayed by a series of snapshots in Figure 29. When the contact between the impactor and the DE region persisted, damage initiated from the tip of the pre-set notch, as shown in Figure 29a, and it propagated upward along a straight direction. This crack propagation behavior is consistent with the experimental observation [86] and the numerical simulation using the FEM [88]. This crack pattern was a mode-I fracture at the macroscale. Furthermore, it can be observed that there was a conical damage pattern emerging at the impact point. This damage pattern initiated upon the impact and gradually propagated in a cone form when the contact between the impactor and DE region persisted, and it stopped until the release of contact.

**Figure 30:**
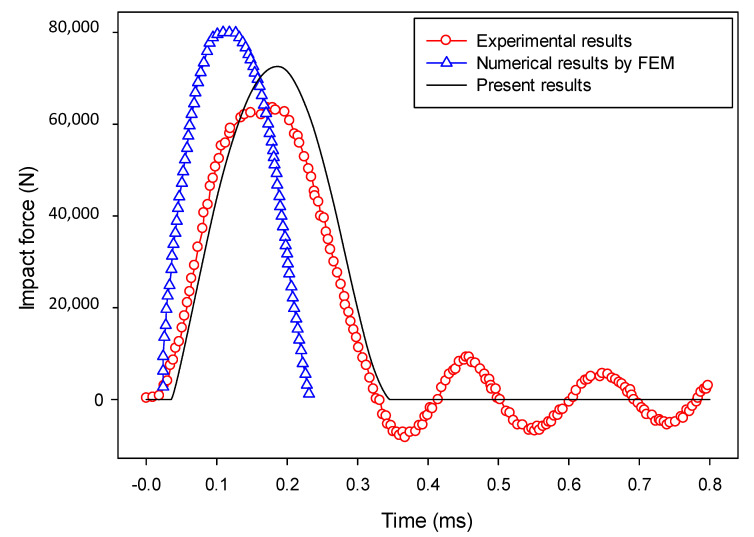
Impact force versus time (impact velocity = 1.76 m/s).

The computed impact force versus time is compared to the experimental results [86] and the numerical results from the FEM (based on continuum damage mechanics) [88] in Figure 30, and the peak force and curve shape were the two most important aspects for the evaluation of the proposed model. It should be mentioned that the penalty factor 0.5 was used, calibrated as seen in Reference [40]. As can be seen in Figure 30, the numerical results agree with the experimental results, although the peak value was slightly larger. Compared with the numerical results using the FEM [88], based on continuum damage mechanics, the results from the proposed model are in better agreement with the experimental results [86]. Note that, due to the difficulty in exact experimental measurement, there were some oscillations appearing in the experimental results in the post-impact stage (after about 0.35 ms). However, these oscillations gradually diminished and tended to converge to the numerical results obtained by the proposed model.

**Figure 31:**
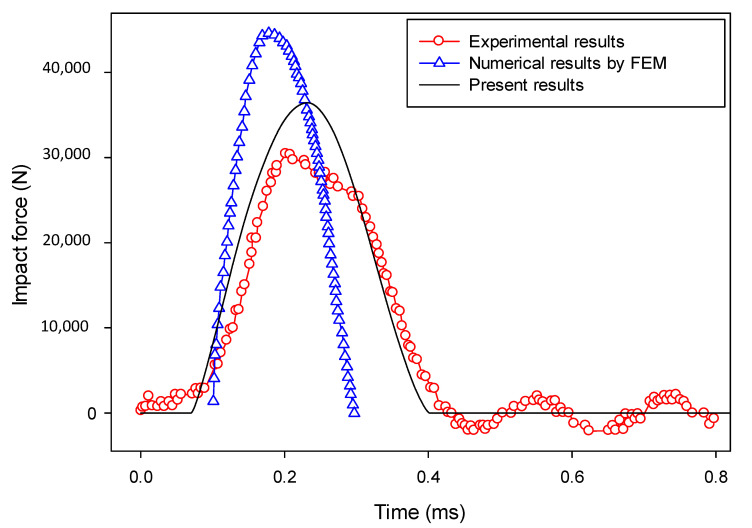
Impact force versus time (impact velocity = 0.881 m/s).

A numerical case with an initial impact velocity of 0.881 m/s was also modeled, and the computed impact force–time relationship from the proposed model was compared with that from the experiment [86] and the numerical analysis using the FEM associated with continuum damage mechanics [88]. It can be found from Figure 31 that the current numerical results agree much better with the experimental results [86] than those obtained from the FEM [88].

**Figure 32:**
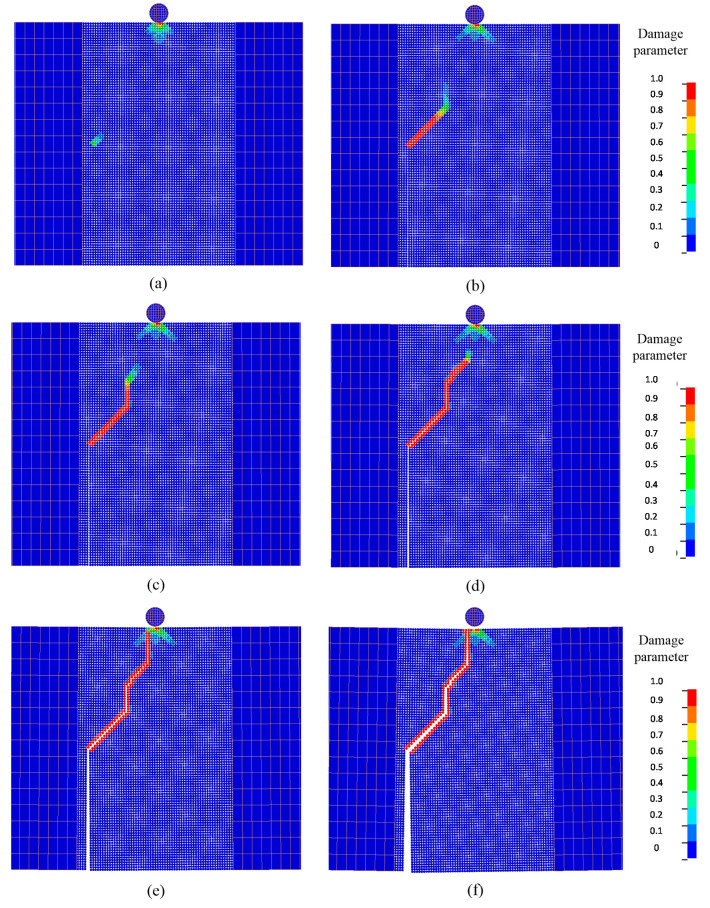
Evolutions of crack and damage in the concrete beam with an initial offset notch: (**a**) t=0.15 ms; (**b**) t=0.24 ms; (**c**) t=0.28 ms; (**d**) t=0.37 ms; (**e**) t=0.57 ms; (**f**) t=0.99 ms.

To show the mixed-mode fracture behavior at macroscale, the initial notch was pre-set to 28 mm left of the mid-span. The initial velocity for this numerical case was 1.76 m/s. A series of snapshots of computed fracture pattern at different times are shown in Figure 32. Damage initiated from the tip of the pre-set notch and initially propagated with an inclined angle. This indicates that the mixed-mode behavior dominated at this stage. Then, cracks moved vertically, indicating that mode-I dominated. After a few transitions of moving direction, cracks propagated to the impact point. The crack propagation behavior which initiated from the notch tip to the area around the loading point agrees with the physical observations [85,89].

## 8. Conclusions

A particle-based cohesive crack model was developed for the connective DE model to model the fracture process of brittle and quasi-brittle materials so as to formulate the material transition from a solid phase to a particulate phase. Because of the particle characteristics of the connective DE model, the cohesive crack model was constructed at inter-particle bonds in the connective stage of the model at microscale. A potential formulation was adopted for the CZM, and a linear softening relation was employed for the traction–separation law upon fracture initiation. The criteria of both the fracture initiation and the fracture propagation were constructed in the displacement space. A damage parameter was used to record damage state and, thus, the fracture process was irreversible. An important feature of this cohesive crack model is that the elastic connective stage of the lattice discrete element model was used as the initial loading stage of the CZM, rather than specifying an artificial loading stiffness as often used by conventional intrinsic CZMs when incorporated into the FEM. This particle-based CZM bridges the microscopic gap between the DE connective model and the DE contact model, and it is suitable to describe the material separation process from solids to particulates.

The proposed model was validated by a number of numerical examples, including standard fracture tests of mode-I, mode-II, and mixed-mode I and II. The close agreement between numerical results and the analytical solution confirmed the effectiveness of the proposed particle-based cohesive crack model in modeling the mixed-mode fracture process. The proposed model was also applied to a notched concrete beam subjected to an impact loading. The fracture processes of both mode-I and mixed-mode I and II were reproduced, and crack propagation was consistent with reality. Furthermore, the impact force obtained from the proposed model was in good agreement with the experimental result and more accurate than the result obtained from the FEM, which employed a continuum damage model.

A limitation of the proposed model is that it can only describe the fracture process of homogeneous and isotropic solids, because the DE connective model can only represent isotropic materials. However, this limitation may not compromise the application of the model in modeling the mixed-mode fracture process of a large range of brittle and quasi-brittle materials, such as glass and ceramics. The DE connective model and the particle-based cohesive crack model could be further extended to anisotropic materials in future work.

## Figures and Tables

**Table 1:** Transition procedures for discrete element (DE) particle pairs.

Initial: All Pairs Being Connective
If (connective and $\psi^{*}\geq 1.0$) then connective 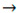 cohesive else if (cohesive and $\lambda^{*}\geq 1.0$) then cohesive 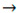 contact end if

**Table 2:** Computational procedures of the interaction force between a particle pair.

If (Connective) then
Use Equations (1) and (5) to calculate internal force $f^{\mathrm{int}}$ else if (cohesive) then
Use Equations (25), (26) and (29) to calculate cohesive force $f^{\mathrm{coh}}$ else if (contact) then
Use Equations (34) and (39) to calculate contact force $f^{\mathrm{con}}$ end if

## Nomenclature

Symbol	Description
${\hat{f}}^{\mathrm{int}}$: (${\;\hat{f}}_{n}$, ${\hat{f}}_{s1}$, ${\hat{f}}_{s2}$)	Interaction force vector and its components in local coordinates.
δ: ($\delta_{n},{\;\delta }_{s1},{\;\delta }_{s2}$)	Relative displacement vector and its components in local coordinates.
K: ($k_{n}$, $k_{s1}$, $k_{s2}$)	Spring stiffness and its components of the normal and two shear spring stiffness.
E, $\;\tilde{E}$	Young’s modulus and equivalent Young’s modulus.
$\tilde{G}$	Equivalent shear modulus.
ν	Poisson’s ratio.
r, $\tilde{r}$	Particle radius and equivalent radius.
n, $s_{1}$, $s_{2}$	The unit base vectors of the local coordinate system to be expressed in global coordinates.
$n_{1}$, $n_{2}$, $n_{3}$	Components of n.
$s_{1}^{1}$, $s_{2}^{1}$, $s_{3}^{1}$	Components of $s_{1}$.
ϕ	Transformation matrix from the global frame to the local frame.
$f^{\mathrm{int}}$, $m^{\mathrm{int}}$	Internal force and associated moment.
$\bar{r}$	The effective radius vector.
$x_{i}$	Position vector of particle *i.*
T: ($T_{n}$,$T_{s1}$,$T_{s2}$)	Traction vector and its components in local coordinates.
A	Effective area.
$\delta_{n0}$, $\delta_{s0}$	Critical values of the relative displacement in the normal and shear directions at the elastic limit.
$T_{n0}$, $T_{s0}$	Material strengths in the normal and shear directions.
$\sigma_{c}$	Material tensile strength.
λ	Non-dimensional scalar of the effective displacement jump.
$\delta_{n}$, $\delta_{s}$	Opening and shear separation components.
$\delta_{\mathrm{nc}}$, $\delta_{\mathrm{sc}}$	Critical values for opening mode and shear mode at complete cracking points.
$\lambda_{\mathrm{cr}}$	Critical value of the scalar at the fracture initiation point.
Φ(λ)	Potential function.
$G_{c}$	Critical energy release rate.
𝒹	Damage index.
𝓇	Loading ratio.
${\hat{f}}^{\mathrm{coh}}$, $f^{\mathrm{coh}}$	Cohesive force vectors in local and global coordinates.
$m^{\mathrm{coh}}$	Moment due to cohesive force in global coordinates.
$G_{Ic}$, $G_{\mathrm{II}c}$	Critical energy release rates of the opening mode and shear mode.
ψ	Non-dimensional scalar.
$f^{\mathrm{con}}$	Contact force vector.
κ	A user-defined penalty factor.
$\tau^{\mathrm{con}}$	The period of persistent contact.
$v_{c}$	Relative velocity $v_{c}$ at contact point c.
$v_{i}$, $\omega_{i}$	Translational and angular velocities.
m, I	Particle mass and moment of inertia.
$f^{\mathrm{ext}}$	External force.
m	Moment induced from all forces.
$N_{i}$	The number of surrounding particles.
L *,* B *,* h	Length, breadth, and half height of the beam.
$a_{0}$, a	Initial and current crack lengths.
𝓘	Second moment of area.
$\chi_{I}$, $\chi_{\mathrm{II}}$	Correction factors for mode-I and mode-II fracture problems.
Γ	Elastic modulus correction parameter.
Δ	Deflection.
P	Load.
C	Loading arm.
β	Mixed-mode ratio.
